# A critical role of RBM8a in proliferation and differentiation of embryonic neural progenitors

**DOI:** 10.1186/s13064-015-0045-7

**Published:** 2015-06-21

**Authors:** Donghua Zou, Colleen McSweeney, Aswathy Sebastian, Derrick James Reynolds, Fengping Dong, Yijing Zhou, Dazhi Deng, Yonggang Wang, Long Liu, Jun Zhu, Jizhong Zou, Yongsheng Shi, Istvan Albert, Yingwei Mao

**Affiliations:** Department of Neurology, The First Affiliated Hospital of Guangxi Medical University, Nanning, Guangxi Province 530021 China; Department of Geriatrics, The 303 Hospital of Chinese People’s Liberation Army, Nanning, Guangxi Province 530021 China; Department of Biology, Pennsylvania State University, University Park, PA 16802 USA; Department of Biochemistry and Molecular Biology, Pennsylvania State University, University Park, PA 16802 USA; Department of Microbiology & Molecular Genetics School of Medicine, University of California, Irvine, CA 92697 USA; Department of Emergency, Guangxi Zhuang Autonomous Region People’s Hospital, Nanning, Guangxi Province 530021 China; Department of Neurology, School of Medicine, Renji Hospital, Shanghai Jiaotong University, Shanghai, 200127 China; Department of Chemistry and Biology, College of Science, National University of Defense Technology, Changsha, 410073 China; Systems Biology Center, National Heart, Lung, and Blood Institute, NIH, Bethesda, MD 20892 USA; Center for Molecular Medicine, National Heart, Lung, and Blood Institute, NIH, Bethesda, MD 20892 USA

**Keywords:** RBM8a, Exon junction complex, Neurodevelopment, Neural progenitor cells, Psychiatric disorders, Nonsense mediated mRNA decay

## Abstract

**Background:**

Nonsense mediated mRNA decay (NMD) is an RNA surveillance mechanism that controls RNA stability and ensures the speedy degradation of erroneous and unnecessary transcripts. This mechanism depends on several core factors in the exon junction complex (EJC), eIF4A3, RBM8a, Magoh, and BTZ, as well as peripheral factors to distinguish premature stop codons (PTCs) from normal stop codons in transcripts. Recently, emerging evidence has indicated that NMD factors are associated with neurodevelopmental disorders such as autism spectrum disorder (ASD) and intellectual disability (ID). However, the mechanism in which these factors control embryonic brain development is not clear.

**Result:**

We found that RBM8a is critical for proliferation and differentiation in cortical neural progenitor cells (NPCs). RBM8a is highly expressed in the subventricular zone (SVZ) of the early embryonic cortex, suggesting that RBM8a may play a role in regulating NPCs. RBM8a overexpression stimulates embryonic NPC proliferation and suppresses neuronal differentiation. Conversely, knockdown of RBM8a in the neocortex reduces NPC proliferation and promotes premature neuronal differentiation. Moreover, overexpression of RBM8a suppresses cell cycle exit and keeps cortical NPCs in a proliferative state. To uncover the underlying mechanisms of this phenotype, genome-wide RNAseq was used to identify potential downstream genes of RBM8a in the brain, which have been implicated in autism and neurodevelopmental disorders. Interestingly, autism and schizophrenia risk genes are highly represented in downstream transcripts of RBM8a. In addition, RBM8a regulates multiple alternative splicing genes and NMD targets that are implicated in ASD. Taken together, this data suggests a novel role of RBM8a in the regulation of neurodevelopment.

**Conclusions:**

Our studies provide some insight into causes of mental illnesses and will facilitate the development of new therapeutic strategies for neurodevelopmental illnesses.

**Electronic supplementary material:**

The online version of this article (doi:10.1186/s13064-015-0045-7) contains supplementary material, which is available to authorized users.

## Background

Autism spectrum disorder (ASD) is a neurodevelopmental disorder characterized by impaired social and communication skills, repetitive, stereotyped behaviors, and often intellectual and cognitive impairments. Intellectual disability (ID) affects roughly 2–3 % of people worldwide [1], and is commonly found in autistic patients. ID typically manifests with an early onset of cognitive impairment. According to The Diagnostic and Statistical Manual of Mental Disorders V (DSM-V), ID is defined by significant deficits in three areas of adaptive behaviors (i.e. conceptual skills, self-help skills, interpersonal skills). Patients with ID normally have the IQ score approximately two standard deviation below the general population, i.e. IQ < 70. Children with ID learn more slowly than a normal child in terms of language, social skills, and the ability to take care of personal needs, such as dressing and eating. Moderate mental retardation (IQ from 35 to 49) is almost always noticeable during the first years of life.

Recently, mutations in a factor involved in nonsense-mediated mRNA decay (NMD), Upf3b, have been found in patients with X-linked ID, autism and early onset schizophrenia [1–3], suggesting an important role for NMD in regulation of neuronal gene expression and mental health. Increasing evidence shows that mutations in NMD factors contribute to neurodevelopmental disorders. In particular, a core exon junction complex (EJC) factor, RBM8a (located in the 1q21.1 chromosome region) is associated with ID, ASD [4], schizophrenia (SCZ) [5], and microcephaly [6]. Duplication of the 1q21.1 region is strongly associated with autism [6]. Moreover, RBM8a loss-of-function causes thrombocytopenia-absent-radius (TAR) syndrome [7] which is often comorbid with ID [8]. Other brain dysfunctions, including psychosis, agenesis of the corpus callosum, and hypoplasia of the cerebellar vermis, are present in TAR patients [8, 9]. In addition, rare mutations on Upf1 have been found in patients with ASD and SCZ [10]. Recently, a mutation in eIF4A3 gene leads to Richieri-Costa-Pereira syndrome with learning disability [11].

NMD was originally identified as an RNA surveillance mechanism that degrades mRNAs possessing a premature termination codon (PTC). The EJC, which consists of four core proteins (eIF4AIII, BTZ, MAGOH, and RBM8a), is essential for NMD [12]. The EJC core serves as a platform to recruit other peripheral proteins, such as Upf1/2/3X [13] and SMG1/5/6/7 [14], to form the SURF complex [15], which breaks down mutated transcripts before translation [13, 14, 16]. Intriguingly, besides RNA surveillance, NMD also regulates the abundance of normal mRNAs [17–19]. Alternative branches of NMD have been discovered [20–22], arguing that different NMD factors may control distinct groups of transcripts instead of the same pool of mRNAs, thereby modulating unique functions [23–32]. To support this notion, several studies have revealed common and unique transcripts regulated by several NMD factors [33–38]. These findings support the hypothesis that NMD plays an important role in the control of gene expression involving a variety of biological processes [23, 24, 26–31, 34, 39–41].

There has also been increasing evidence that NMD proteins play a role in neurodevelopment. Magoh haploinsufficiency was found to cause microcephaly in mice, in addition to disorganized cortical layers, ectopic differentiation, and mitotic spindle defects [30, 42]. Upf3b has also been found to play a role in neurodevelopment. Loss of function of Upf3b leads to an increased number of NPCs, paired with a decrease in differentiation. Upf3b is also instrumental in neural maturation, as loss of Upf3b results in subtle neurite outgrowth defects [43]. Further evidence supporting the notion that NMD proteins are involved in development is a study conducted on Upf1, and its role in proliferation and differentiation. Upf1 was found to promote the stem like state of cells, and is downregulated to promote differentiation. Specifically, Upf1 promotes proliferation at the transition from G1 of the cell cycle to the S phase. These changes were found to be mediated by the TGF-β pathway [35]. Additionally, studies in *Drosophila melanogaster* have shown that loss of mago (homologue of Magoh), prevents EGF signaling via downregulation of the MAPK pathway. This modulation of the MAPK pathway is dependent of EJC factors, Mago, RBM8a, and eIF4AIII. The reduction of MAPK signaling is due to alternative splicing of MAPK, while transcriptional levels and RNA stability of MAPK is maintained [40, 41]. The EJC’s regulation of alternative splicing is not just specific to MAPK, but also includes other long, intron containing genes [40, 41].

RBM8a, an EJC factor, is a ribonucleoprotein with an RNA binding motif that preferably binds to mRNAs during splicing [44]. Its role in NMD has been extensively studied [45, 46]. However, specific cellular functions mediated by RBM8a have not been well characterized. In addition to controlling mRNA stability, RBM8a also regulates mRNA splicing [47, 48] and translation [49]. RBM8a was found in an mRNA export machinery through importin13 [44]. Localization of some specific mRNAs in *Drosophila melanogaster* determines its embryonic polarity. RBM8a and other EJC factors anchor *Oskar* mRNA at the posterior pole of the oocyte and regulates *Oskar* localization, and thereby control oocyte maturation [50, 51]. In addition, RBM8a associates with the C-terminal domain of Stat3 and regulates Stat3 transcriptional activity through modulation of tyrosine phosphorylation [52]. As these studies used special cell types, it is still unclear whether these RBM8a-mediated functions can be observed in other cell types.

Despite the genetic studies strongly implicating that NMD is crucial for neural functions [53], very little is known about its specific effects on the neural development. To determine the role of NMD in the central nervous system, we first examined the function of RBM8a in the embryonic NPC. Our data supports a positive role of RBM8a in regulating NPC proliferation. Loss-of-function of RBM8a leads to abnormal NPC proliferation and differentiation. To determine the underlying mechanism, an unbiased RNAseq of the transcriptome of SY5Y cells overexpressing RBM8a, has revealed multiple important functions regulated by RBM8a that have not been previously reported.

## Results

### RBM8a is highly expressed in neural progenitors during brain development

**Fig. 1:**
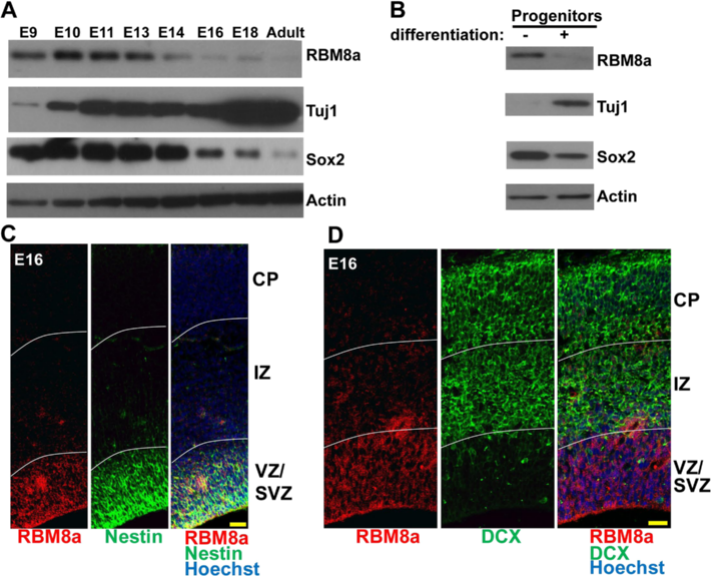
RBM8a expression in embryonic brain. **a** Expression of RBM8a in embryonic brain from E9 to adult. **b** RBM8a is highly expressed in NPCs but reduced in differentiated neurons. **c** Co-immunostaining of RBM8a (red) and Nestin (green) in E16 embryonic cortex. Scale bar = 20 μm. **d** Co-immunostaining of RBM8a (red) and DCX (green) in E16 embryonic cortex. Scale = 20 μm. Noticeably, Nestin + NPCs express high RBM8a level in the VZ/SVZ, but low in the IZ and the CP. CP, cortical plate; IZ, intermediate zone; VZ/SVZ, ventricular zone/subventricular zone

To test the role of RBM8a in brain development, we first examined its expression in mouse brains from E9 to adult. Early embryonic brains (E9-E14) express a significantly higher level of RBM8a when NPCs are actively proliferating (E10-E13), and then have decreased expression at E14 when NPCs begin to differentiate (Fig. 1a). Interestingly, the NPC marker, Sox2, exhibits a similar expression pattern, suggesting that RBM8a may share similar functions as Sox2. We next tested whether RBM8a expression changed during primary NPC differentiation. NPCs were isolated from E14 mouse brains and then induced to differentiate for 7 days *in vitro*. RBM8a levels were dramatically reduced after differentiation (Fig. 1b). In the embryonic cerebral cortex, RBM8a is robustly expressed in nestin positive neural progenitors residing in the ventricular zone (VZ)/subventricular zone (SVZ) (Fig. 1c), but reduced in doublecortin (DCX) positive progenitors or immature neurons in the intermediate zone (IZ) and the cortical plate (CP) (Fig. 1d). The striking expression pattern of RBM8a suggest that it may play an important role in progenitor proliferation and differentiation.

### RBM8a promotes neural progenitor cell proliferation

As NMD has been implicated in cell proliferation [54, 55], we generated two short hairpin RNAs (shRNAs) against the endogenous mouse RBM8a gene. The two shRNAs specifically knocked down RBM8a in CAD cells, a well-established mouse neuroblastoma cell line, which can proliferate and differentiate (Additional file 1A). The specificity of RBM8a staining was verified using CAD cells transfected with either control or RBM8a shRNA (Additional file 1B), in which CAD cells expressing RBM8a shRNA (GFP positive) showed lower RBM8a staining compared to the GFP-negative cells in the same field. Based on its expression in neural progenitors, we next looked into a potential role for RBM8a in cell proliferation *in vitro*. When RBM8a was knocked down in CAD cells, BrdU labeling (0.5 h pulse) and mitotic index were dramatically decreased by more than 3-fold (Additional file 2A and B). As duplication of 1q21 region has also been implicated in neurodevelopmental disorders, the reduced proliferation by RBM8a knockdown encouraged us to pursue the reciprocal RBM8a gain-of-function experiment. Remarkably, overexpression of full length human RBM8a stimulates a 2-fold increase in cell proliferation, 2-fold increase in both BrdU incorporation and mitotic index (Additional file 2C and 2D). Thus, RBM8a is required for the normal proliferation and its overexpression promotes proliferation.

**Fig. 2:**
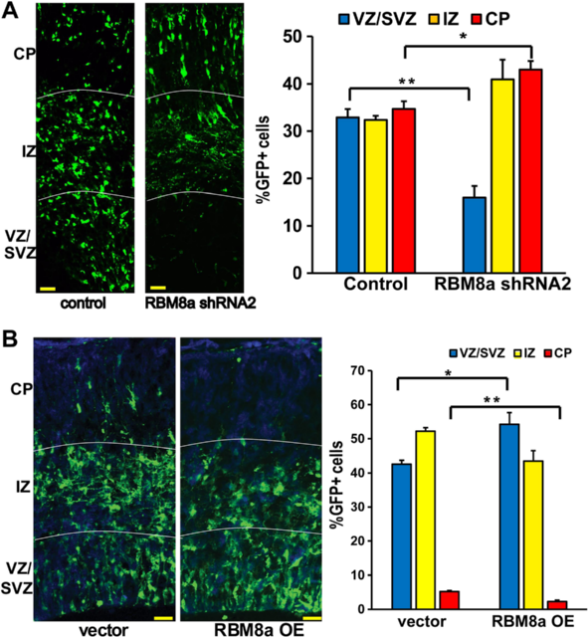
RBM8a regulates cell position in the cortex. **a** Confocal images of E16 brain sections showing cell positioning after *in utero* electroporation of shRNA constructs at E13. The graph shows that knockdown of RBM8a significantly decreased the number of GFP-positive cells in the VZ/SVZ and increased the cell number in the CP. *, *p* < 0.05. **, *p* < 0.01; *n* = 3-4, *t*-test. Scale bar = 20 μm. **b** E16 brain sections show cell positioning after *in utero* electroporation of an overexpression construct at E14. Quantification shows that overexpression of WT-RBM8a significantly decreased the number of GFP-positive cells in the CP. *, *p* < 0.05. **, *p* < 0.01; *n* = 3-4, *t*-test. Scale bar = 20 μm

We next tested how change of RBM8a expression affects NPC behaviors *in vivo. In utero* electroporation was used to deliver RBM8a shRNA2 into E14 embryonic mouse brains (Fig. 2a). RBM8a shRNA and a GFP plasmid were co-delivered into neural progenitors by injection into the ventricle, and application of an electrical current was then used to direct the plasmid DNA into specific populations of progenitor cells. As neural progenitor cells divide and differentiate, newborn neurons migrate from the VZ/SVZ through the IZ and into the developing CP to their final destination where they exist fully mature. We therefore tested the effect of RBM8a on NPC position and proliferation at E16, which is a time of peak neurogenesis. Knockdown of RBM8a from E13 to E16 *in utero* decreased the cell number at the VZ/SVZ, and increased the number of cells migrating into the CP, where differentiated neurons are localized (Fig. 2a). To confirm the migration defect caused by RBM8a knockdown, we electroporated RBM8a shRNA2 at E15, when neuronal migration peaks (Additional file 3A). After harvesting brains at E18, similar migration defects were observed. Conversely, RBM8a overexpression led to a decrease in the cell number migrating into the CP (Fig. 2b), suggesting that RBM8a up-regulation may impair the NPC development process. These results suggest that NPC proliferation is reduced and neuronal differentiation is increased after RBM8a knockdown, whereas RBM8a overexpression suppresses neuronal differentiation.

**Fig. 3:**
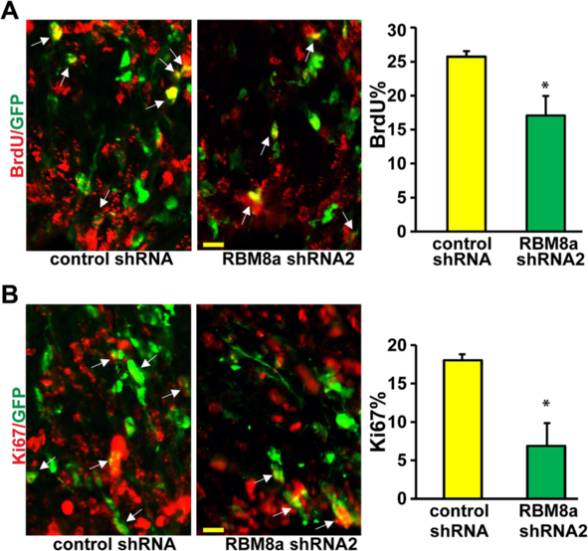
RBM8a knockdown decreases NPC proliferation. **a** RBM8a was knocked down at E13, and pulse labeled with BrdU (100 mg/kg) for 2 h. E16 brain sections were stained for BrdU (red) and GFP (green). Arrowheads indicate GFP-BrdU double-positive cells. Graphs show the percentage of GFP/BrdU double positive cells in each condition. *, *p* < 0.05; *n* = 4. *t*-test. Scale bar =10 μm. **b** E16 brain sections were stained for Ki67 (red) and GFP (green). Arrowheads indicate GFP-Ki67 double-positive cells. Graphs show the percentage of GFP/Ki67 double positive cells in each condition. *, *p* < 0.05; *n* = 4.*t*-test. Scale bar =10 μm

**Fig. 4:**
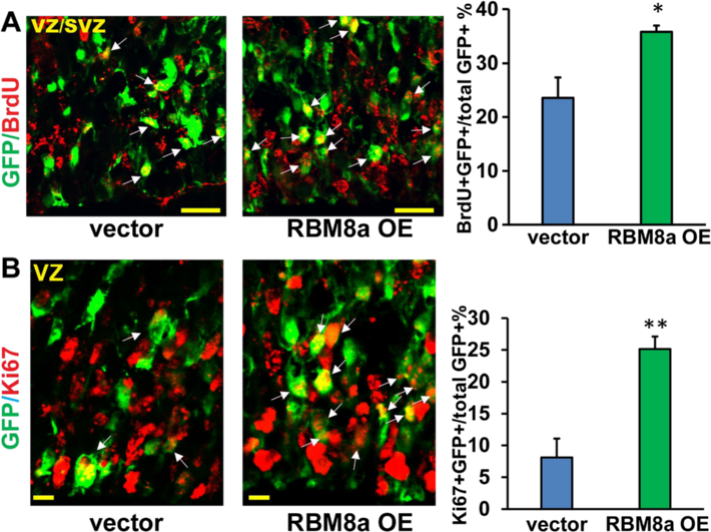
RBM8a overexpression increases NPC proliferation. **a** RBM8a was overexpressed at E14, and E16 brain sections were stained for BrdU (red) and GFP (green). Arrowheads indicate GFP-BrdU double-positive cells. Graphs show the percentage of GFP/BrdU double positive cells in each condition. *, *p* < 0.05; *n* = 3–4. *t*-test. Scale bar = 10 μm. **b** E16 brain sections were stained for Ki67 (red) and GFP (green). Arrowheads indicate GFP-Ki67 double-positive cells. Graphs show the percentage of GFP/Ki67 double positive cells in each condition. **, *p* < 0.01; *n* = 3–4. *t*-test. Scale bar = 10 μm

We hypothesize that RBM8a may regulate cell positioning by affecting NPC proliferation *in utero.* To test this hypothesis, we first knocked down RBM8a at E13 and pulse-labeled embryos with BrdU (10 mg/kg) to label progenitors undergoing DNA replication 2 h prior to collection of the electroporated brains. The E16 brains were subsequently harvested. Staining for BrdU revealed that knockdown of RBM8a significantly decreased the percentage of double positive cells for GFP/BrdU (Fig. 3a), supporting the postulate that RBM8a knockdown expression reduces the number of proliferating NPCs. Consistently, the active cell cycle marker, Ki67, is decreased in the RBM8a knockdown cells (Fig. 3b). Conversely, when RBM8a was overexpressed at E14, we detected an increase of GFP/BrdU double-positive cells (Fig. 4a) at E16. Interestingly, RBM8a overexpression promotes cell division, as more GFP positive cells from overexpression group are positive for Ki67 than from vector control (Fig. 4b). These data suggest that RBM8a is a positive regulator for NPC proliferation.

### RBM8a modulates cell cycle progression and neuronal differentiation

**Fig. 5:**
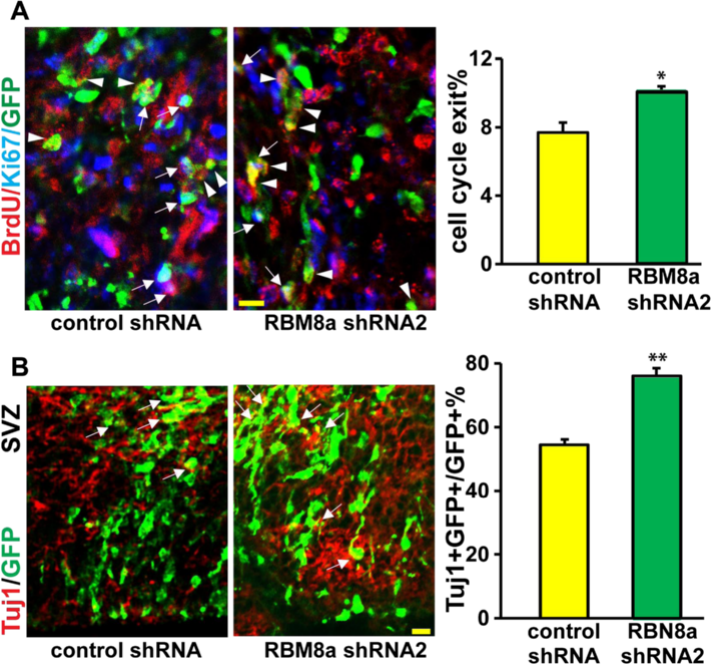
RBM8a knockdown in NPCs causes premature cell cycle exit and neuronal differentiation *in utero*. **a** Control or RBM8a shRNA constructs were electroporated into E13 embryonic brains and BrdU was injected at E15. Mice were sacrificed at E16. The cell cycle exit index is calculated as the percentage of the GFP-positive cells that exited the cell cycle (GFP+ BrdU+ Ki67-) divided by total GFP and BrdU double positive (GFP+ BrdU+) cells. *n* = 3-4, *, *p* < 0.05, *t*-test. Scale bar = 10 μm. White arrows indicate GFP + BrdU + Ki67+ cells. Arrowheads indicate GFP + BrdU + Ki67- cells. **b** Control or RBM8a shRNA constructs were electroporated into E13 embryonic brains and mice were sacrificed at E16. E16 brain section were stained with neuronal marker Tuj1 (red) and GFP (green). Graphs show the percentage of GFP/Tuj1 double positive cells in each condition. **, *p* < 0.01; *n* = 3–4. *t*-test. Scale bar = 10 μm

Our data suggest the cell cycle may be changed by RBM8a silencing. To test this possibility, we examined the cell cycle exit index. RBM8a shRNA constructs were electroporated into E13 mouse brains and BrdU was injected into pregnant dams 2 days later, at E15. Sections from E16 brains were collected and stained using anti-GFP, −BrdU, and -Ki67 antibodies (Fig. 5a). GFP+/BrdU+/Ki67+ cells (arrows) were in S phase at E15 and remain cycling at E16 (Fig. 5a). GFP+/BrdU+/Ki67- cells were in S phase at E15, but exited the cell cycle by E16 (arrowheads). The cell cycle exit index represents the ratio of GFP+/BrdU+/Ki67- to total GFP+/BrdU+ cells. We observed a 32 % increase in cell cycle index in RBM8a shRNA transfected embryonic brains, suggesting that the reduction of proliferating progenitors in RBM8a shRNA treated brains probably results from increased cell cycle exit. To further evaluate the fate of the cells that exited the cell cycle, we immunostained the brain sections for β-III tubulin (Tuj1), a marker for newly generated neurons (Fig. 5b). A significant increase in Tuj1 positive cells was observed with RBM8a shRNA transfected cells (76 ± 2.4 % for shRNA-2) compared to control shRNA transfected cells (54.5 ± 1.7 %) (Fig. 5b, Additional file 3B). Taken together, these results suggest that loss of RBM8a expression causes premature neuronal differentiation at the expense of the progenitor pool.

**Fig. 6:**
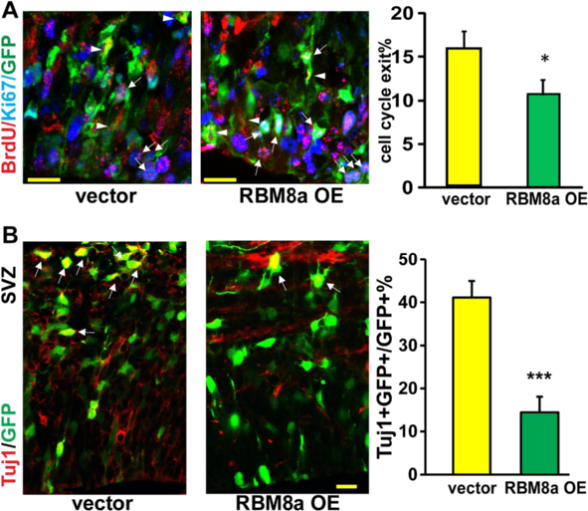
RBM8a overexpression prevents cell cycle exit and neuronal differentiation *in utero*. **a** Control or RBM8a overexpressing constructs were electroporated into E14 embryonic brains and BrdU was injected at E15. Mice were sacrificed at E16. The cell cycle exit index is calculated as the percentage of the GFP-positive cells that exited the cell cycle (GFP+ BrdU+ Ki67-) divided by total GFP and BrdU double positive (GFP+ BrdU+) cells. n = 3-4, *, *p* < 0.05, *t*-test. Scale bar = 10 μm. White arrows indicate GFP + BrdU + Ki67+ cells. Arrowheads indicate GFP + BrdU + Ki67- cells. **b** Control or RBM8a overexpressing constructs were electroporated into E14 embryonic brains and mice were sacrificed at E16. E16 brain section were stained with neuronal marker Tuj1 (red) and GFP (green). Graphs show the percentage of GFP/Tuj1 double positive cells in each condition. ***, *p* < 0.001; *n* = 3–4. *t*-test. Scale bar = 10 μm

As upregulation of RBM8a increases NPC proliferation, we hypothesize that RBM8a overexpression suppresses the cell cycle exit thereby increasing proliferation. Consistently, we observed about 30 % decrease of cell cycle exit when RBM8a level is upregulated (Fig. 6a). To further examine neuronal differentiation of NPCs when RBM8a is overexpressed, brain sections were co-stained with Tuj1 and GFP antibodies. Consistent with increased proliferation of NPCs, upregulation of RBM8a significantly suppresses neuronal differentiation (Fig. 6b, Additional file 3C). Together, these data suggest that an optimal level of RBM8a is essential for proper cell cycle control and NPC differentiation (Figs. 5 and 6).

### Genes regulated by RBM8a

**Fig. 7:**
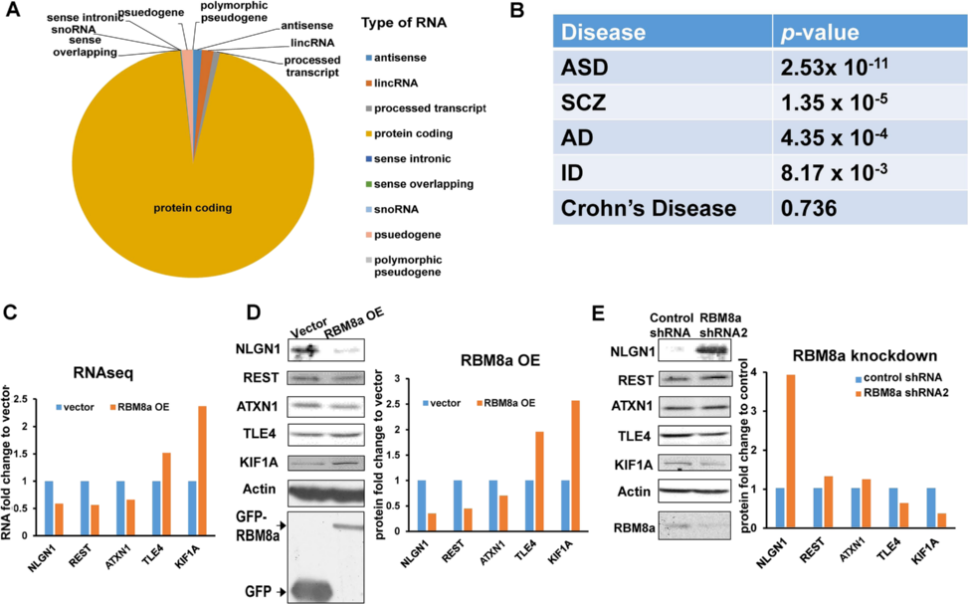
Illumina RNAseq from RBM8a over-expression. **a** RNA types that are changed by RBM8a. **b** Downstream genes regulated by RBM8a were cross referenced with different diseases including AD, ASD, ID, SCZ and Crohn’s diseases. **c** Fold change of RNA level of NLGN1, ATXN1, REST, TLE4, KIF1A from RNAseq. **d** Protein level of NLGN1, ATXN1, REST, TLE4, KIF1A in CAD cells overexpressing RBM8a. Bar graph shows the fold change of protein level normalized to vector expressing cells. **e** Protein level of NLGN1, ATXN1, REST, TLE4, KIF1A in CAD cells silencing RBM8a. Bar graph shows the fold change of protein level normalized to control shRNA expressing cells

Our results have shown that altered expression of RBM8a impedes NPC proliferation (Figs. 3 and 4) and impairs neuronal differentiation (Figs. 5 and 6). Although several cellular functions of RBM8a have been discovered in non-neural tissues [50, 51, 56], it is unknown what molecular targets and signaling pathways are regulated by RBM8a. To identify differential expressing genes regulated by RBM8a, we have established a stable neural SH-SY5Y cell line overexpressing RBM8a *in vitro* (Additional file 4A). The RNASeq platform is capable of identifying relative expression, alternative splicing, novel transcripts and isoforms, RNA editing, and allele-specific expression. RNAs from control and induced cells, in which rRNAs have been removed, were sequenced using the Illumina HiSeq 2500 system. Unbiased RNASeq generates 50 million high-quality paired-reads per sample (at 50 nt) (Additional file 4B and C) and over 95 % of the reads are mapped back to the reference genome. We have found that 7.08 % of transcripts are differentially expressed when RBM8a is overexpressed, which is consistent with other studies [17–19, 57]. Interestingly, the most affected transcripts are protein coding mRNAs (94.7 %) and the others are noncoding RNAs. The three largest groups of noncoding RNAs include lincRNAs (1.57 %), antisense RNAs (1.17 %), and pseudogenes (1.46 %) (Fig. 7a, Additional file 5: Table S1). These genes represent downstream factors that mediate RBM8a functions.

First, we compared RBM8a-regulated genes with existing databases for psychiatric risk genes to determine whether there were significant gene overlaps. Using the hypergeometric analysis we determined that risk genes in ID, Alzheimer’s disease (AD) [58], ASD [59, 60] and SCZ [61–63] but not Crohn’s disease [64], are over-represented in RBM8a downstream gene targets (Fig. 7b, Additional file 6: Table S2), suggesting that genes common in both datasets are not likely due to by chance. To examine the protein level of the RNAseq results in mouse CAD cells, we tested genes involved in autism risk (NLGN1) [65], neurodegeneration (ATXN1) [66], neurogenesis (REST) [67], embryonic development (TLE4) [68], and neuronal migration (KIF1A) [69]. Consistent with the RNA levels from our RNAseq data (Fig. 7c), overexpression of RBM8a in CAD cells also produces corresponding level of proteins (Fig. 7d). In the other hand, knockdown of RBN8a generates reversal level of proteins of these genes in CAD cells (Fig. 7e). Therefore, these results confirm the validity of our RNAseq data and support the theory that RBM8a is involved in increased risk for mental illnesses.

**Fig. 8:**
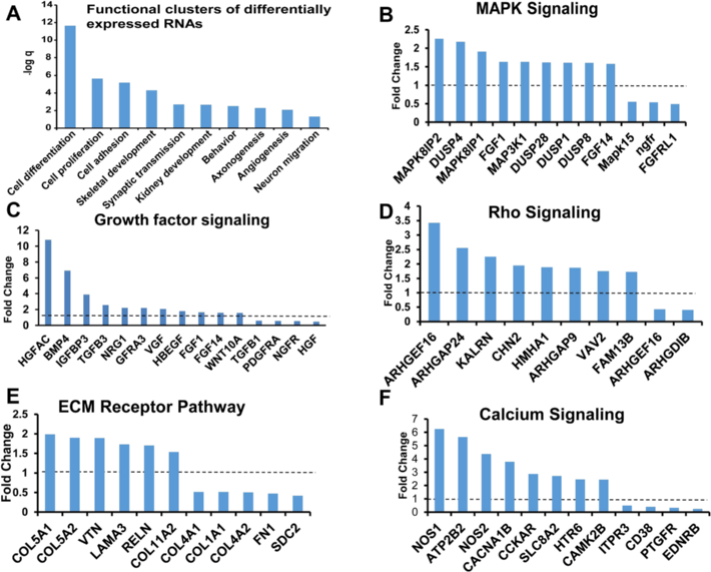
Pathway analysis of genes that are modulated by RBM8a. **a** Major functions regulated by RBM8a. **b** The fold changes of factors in the MAPK pathway. Graphs show the fold changes of mRNA expression over control. **c** The fold changes of genes in growth factors. **d** The fold changes of factors in the Rho pathway **e** ECMs are regulated by RBM8a. **f** The fold changes of factors in the calcium signaling

To analyze the underlying signaling pathways that are regulated by RBM8a, we utilized the DAVID Bioinformatics tool [70]. Based on our dataset of RBM8a regulated genes, DAVID determined that RBM8a regulates genes involved in several processes vital for embryonic brain development (Fig. 8a), including the MAPK pathway (Fig. 8b), growth factor signaling (Fig. 8c), the Rho signaling (Fig. 8d), extracellular matrix (ECM) receptors including integrins and collagens (Fig. 8E), and Ca^2+^ signaling (Fig. 8f). Our data demonstrates that RBM8a regulates many functions during brain development (Fig. 8, Additional file 7: Table S3). Intriguingly, besides genes that are required for neurodevelopment, our data also indicates that RBM8a regulates genes involved in kidney, urogenital system and vasculature development (Additional file 7: Table S3). This is consistent with the fact that TAR patients often develop renal and cardiovascular anomalies [71].

**Fig. 9:**
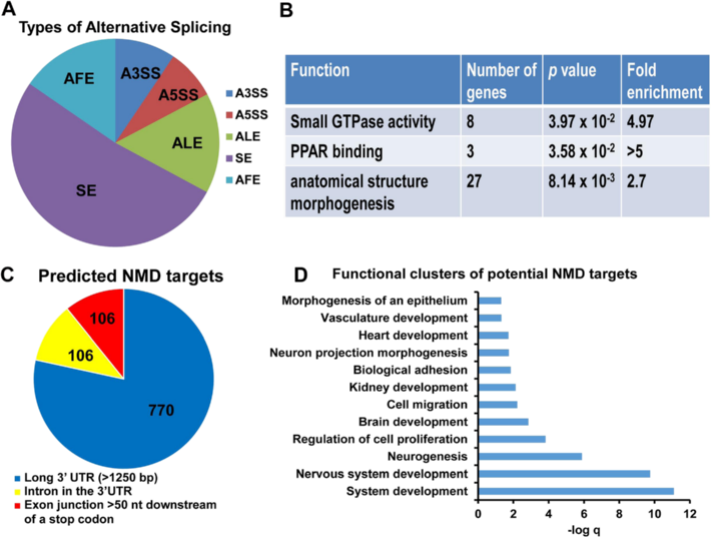
RNAseq analysis of alternative splicing and NMD targets. **a** The percentage of each alternative splicing type found in the dataset, skipped exon (SE), alternative 3′ splice site (A3SS), alternative 5′ splice site (A5SS), alternative last exon (ALE) and alternative first exon (AFE). **b** Functions mediated by alternatively spliced genes. **c** The percentage of differentially expressed RNAs that possess characteristics making it prone to degradation via NMD. **d** Functional clusters based on differentially expressed RNAs that are prone to NMD

Next, since EJC proteins have been implicated in alternative splicing [72], we investigated whether RBM8a overexpression leads to change of differential alternative splicing. Our dataset identified that 371 alternative splicing events in 101 protein coding genes were significantly altered (Fig. 9a, Additional file 8: Table S4). In contrast to hnRNP A1 [73], an alternative splicing regulator, RBM8a overexpression gives rise to fewer alternative splicing events, indicating that while RBM8a does have an effect on alternative splicing, it is not an a global splicing regulator. Interestingly, the alternatively spliced protein coding genes are significantly enriched in ASD risk genes (Additional file 9: Table S5). Interestingly, these genes regulates small GTPase activity, PPAR binding and cell morphogenesis (Fig. 9b).

As RBM8a is involved in NMD, we wanted to ascertain whether or not our differentially expressed RNAs are NMD targets. Characteristics of RNAs targeted by NMD are long 3′ UTRs (greater than 1250 bp), an intron in the 3′UTR, or an exon junction >50 nt downstream of a stop codon [74]. We assessed our list of differentially expressed RNAs to determine how many exhibit characteristics that would make the RNA prone to degradation via NMD. 814 genes out of 1788 differentially expressed genes were determined to possess features that make the RNA susceptible to NMD (Fig. 9c, Additional file 10: Table S6). We were also interested in what functions are mediated by the differentially expressed RNAs targeted by NMD. We found that this subset of RNAs regulates many processes associated with neural development (Fig. 9d). Seeing as we previously determined that our differentially expressed RNAs were enriched for neurological disease risk gene, we tested if this phenomenon held true for those RNAs targeted by NMD. In fact, we did see that our dataset was enriched with risk genes for ASD, SCZ, AD, and ID, but not Crohn’s disease (Additional file 11 and Additional file 12: Table S7). Interestingly, among the 97 genes that are involved in ASD risks, many modulate neurodevelopment and synaptic function (Additional file 11B).

## Discussion

During early brain development, NPCs in the SVZ/VZ can self-renew and differentiate into neurons. The development of the nervous system is a delicate balance of proliferation and differentiation. Enough NPCs need to remain NPCs to give rise to a sufficient number of cells to support the organism, while some NPCs need to differentiate in order to build the nervous system, so the organism can support itself. In this study, we demonstrated that RBM8a plays a key role in NPC proliferation and differentiation. First, RBM8a is highly expressed in the SVZ of early embryonic cortex. To confirm the role of RBM8a in early neurogenesis, *in utero* electroporation using RBM8a shRNA in mouse embryonic brains has demonstrated that knockdown of RBM8a leads to decreased number of cells in the VZ/SVZ, and more cells in the CP (Fig. 2a). This suggests that fewer NPCs are remaining as NPCs, and more NPCs are differentiating and migrating to the CP. Conversely, when RBM8a is overexpressed, more NPCs are remaining in the VZ/SVZ and proliferating, while less cells are present in the CP, suggesting that fewer cells are differentiating and migrating to the CP (Fig. 2b). Moreover, overexpression of RBM8a suppresses cell cycle exit and keeps cortical NPCs in proliferative state. The loss and gain of function data indicate that RBM8a promotes proliferation, while suppressing differentiation. Therefore, we conclude that RBM8a stimulates embryonic NPC proliferation and suppresses neuronal differentiation, indicating that RBM8a positively regulates NPC proliferation.

NMD factors are known to control embryogenesis. Upf2 is involved in development of hematopoietic stem cells [75]. The first study of Magoh mutant mice demonstrated the important role of EJC in NPC division [30]. Recently, Upf1 has been shown to be sufficient to promote neural differentiation and inhibit NPC proliferation *in vitro* and low vertebrate animal [35]. Although RBM8a participates in the NMD pathway and controls mRNA splicing and degradation [15, 76, 77], the role of RBM8a in the embryonic brain is unclear. Our data strongly supports a vital role of RBM8a in the maintenance of a NPC pool in the VZ/SVZ. The first evidence is that the expression of RBM8a is high in the Nestin-positive progenitors of the developing cortex (Fig. 1c). Second, RBM8a expression level is decreased when NPCs differentiate into neurons in the CP (Fig. 1), suggesting the expression level of RBM8a critically determine the stemness of NPCs. Consistent with this notion, knockdown of RBM8a significantly decreases NPC proliferation and promotes neuronal differentiation (Figs. 3 and 5). Additionally, RBM8a controls the cell cycle of NPCs. Thus, our data is consistent with recent studies that the NMD activity is important in maintaining the balance of proliferation and differentiation in NPCs [30, 35, 55].

Our knockdown data is consistent with a recently published paper that illustrated that RBM8a haploinsufficiency causes ectopic neuronal differentiation, and disrupts the balance between proliferation and differentiation [78]. However, our data suggests that RBM8a overexpression tips the balance between proliferation and differentiation in the opposite direction as RBM8a knockdown, which they did not detect. This difference may most likely be attributed to the promoter used to drive RBM8a expression. Different promoters can lead to a different level of RBM8a expression, which might explain the variation of phenotype.

Several mechanisms may underlie the loss-of-function phenotypes of NMD in NPCs. Magoh regulates mitosis of NPCs through LIS1 [30]. To uncover the underlying mechanisms regulated by RBM8a, genome-wide RNAseq identifies potential substrates of RBM8a in the brain, which have been implicated in neurogenesis and plasticity. However, our RNA-seq analysis has indicated that Lis1 is not differentially expressed when RBM8a is overexpressed, suggesting that Lis1 might not be a downstream target of RBM8a. Although Magoh and RBM8a form a heterodimer in conjunction with their role in NMD, increasing evidence has illustrated that the two proteins may have differential roles in aspects independent of NMD. In humans, TAR syndrome occurs when there is a compound mutation in RBM8a (deletion of the 1q.21.1 region in one allele, and a mutation in the RBM8a gene in the other allele) [7]. This disorder, however, does not seem to involve Magoh. Genetically this disorder is attributed to mutations in the RBM8a gene, but not Magoh. Additionally, it has been shown that RBM8a shuttles between the nucleoplasm and cytoplasm independent of Magoh, further supporting the theory that RBM8a and Magoh have independent functions, in addition to their role in NMD [79]. RBM8a is also known to positively regulate TNF-α induced IL-6 expression in HeLa cells, which Magoh is not involved in [80]. Together, these studies suggest that RBM8a and Magoh each have separate functions from each other, in addition to their collaborative role in NMD.

RBM8a may play a regulatory role in several stages during brain development. Our data indicates that alteration of RBM8a expression impairs neuronal migration (Fig. 2). ECM proteins including integrin and collagens are crucial for neurogenesis [81]. ECMs are differentially expressed when RBM8a is overexpressed (Fig. 8e), suggesting that RBM8a regulates neuronal migration through ECM proteins. Previously it has been shown that alteration of RBM8a level in the adult dentate gyrus affects anxious behaviors in mice [55]. Consistently, we have found that RBM8a overexpression significantly affects genes involved in calcium signaling, which is required for neurotransmission. This change in calcium signaling may explain the abnormal behaviors seen in mice who have increased RBM8a expression in the dentate gyrus. This sequencing data further demonstrates that RBM8a regulates multiple processes during brain development. Functionally, we were also able to determine what biological functions are significantly mediated by the RNAs regulated by RBM8a. Interestingly, these functional categories include neuronal differentiation, cell proliferation, biological adhesion, and cell migration, which is consistent with our *in vivo* data. Also of note, is that there is a significant number of RNAs in our dataset that regulate skeletal development and vascular development. This is particularly interesting, since TAR syndrome, which is caused by a compound mutation in RBM8a, is characterized by missing radii bones (skeletal development) and low blood platelet counts (vasculature). Additional enriched functions seem to surround developmental and neurological processes (Additional file 7: Table S3).

Since RBM8a play a key role in NMD, we identified that 814 out of 1788 differentially expressed RNAs possessed characteristics that would make the RNA prone to degradation via NMD (long 3′UTR, intron in the 3′UTR or exon junction > 50 nt downstream of a stop codon). Functions mediated by these genes include many developmental processes such as neurogenesis, cell motility and communication, and the development of the nervous system (Fig. 9). This data is consistent with the phenotypes seen in our *in vitro* and *in vivo* experiments for knockdown and overexpression of RBM8a. Another interesting function regulated by these genes is renal development, which is disrupted in patients with TAR syndrome [71].

Interestingly, ASD- and SCZ-risk genes are highly representative in RBM8a-mediated transcripts. After further analyzing these differentially expressed RNAs targeted by NMD by cross-referencing them with risk genes for neurological disease, ASD, SCZ, AD, and ID risk genes but not our control, Crohn’s disease were highly represented in our sample (Additional file 12: Table S7). ASD risk genes were the most enriched (*p* = 4.12 × 10^−9^). These overlapping genes were found to regulate processes associated with neurogenesis, cell communication and synapse organization, among other functions.

We also used our RNAseq data to assess the effect of RBM8a overexpression on alternative splicing, seeing how NMD proteins have been previously determined to regulate alternative splicing [82]. There were 314 alternative splicing events found in our dataset, spanning a variety of types of alternative splicing. The most prominent type of alternative splicing was skipped exon (61.4 %), followed by alternative last exon (18.5 %). 9.24 % of alternative splicing events were alternative 5′ splicing site, while 11.15 % were alternative 3′ splicing site. This data indicates that RBM8a does seem to have an effect on alternative splicing even though the effect is small. The genes that were determined to be alternatively spliced were enriched for ASD risk genes, but not SCZ, AD, ID, or Crohn’s disease risk genes (Additional file 9: Table S5). This data supports the hypothesis that RBM8a may be involved in the pathogenesis of ASD through an increase in alternative splicing of ASD risk genes.

Throughout all of our analyses of our RNAseq data, developmental processes, specifically neurogenesis, have remained consistent among all of our datasets (differentially expressed RNAs, cross-reference with ASD, NMD targets, alternatively spliced genes etc.). These processes were those found to be abnormal in our overexpression and knockdown experiments, indicating an important role for RBM8a in neural development, and the risk for neurological and psychiatric disease.

Studies of RBM8a and other NMD factors in embryonic brain development may help understand the pathophysiology of several neurodevelopmental disorders such as ID, ASD and SCZ. Upf3b gene has been implicated in ID, ASD and SCZ. In the past several decades, tremendous advancement was made in identification of genetic components causing ID, ASD, and SCZ. Chromosomal abnormalities such as copy number variations (CNVs) including both duplications and deletions are enriched in many patients with ID, ASD and SCZ [4]. Some of these CNVs, including 1q21 [6], are significantly associated with all three disorders. As RBM8a is localized in 1q21 region, our studies provide an insight on causes of mental illnesses and will facilitate the development of new targets for neurodevelopmental illnesses.

## Conclusion

Taken together, our data demonstrate a critical role of RBM8a in regulation of early neurodevelopment. RBM8a stimulates embryonic neural progenitor proliferation and suppresses neuronal differentiation. Unbiased RNA-seq analysis reveals that RBM8a regulates many risk genes involved in neurodegenerative/neuropsychiatric diseases and multiple important functional processes that are important for early neurodevelopment. Our studies provide a deeper insight on causes of neurological illnesses.

## Methods

### Mice

Wild type male and female C57/B6N mice were obtained from Taconic. C57BL/6 N male mice were housed (2–4 mice per cage) in a room with a light/dark cycle at 12 h interval (lights on at 7:00 am), and provided *ad libitum* access to food and water. All procedures on mice were reviewed and approved by The Pennsylvania State University IACUC committee, under IACUC protocol, 44057, to Yingwei Mao.

### Cell culture

Mouse CAD and human 293 T, SH-SY5Y cells were cultured in DMEM medium containing 10 % FBS, L-glutamine, and penicillin/streptomycin. Primary NPCs were isolated from E14 mouse embryos and cultured as described previously [83]. The cortex from 6 to 8 mouse brains at E14 were dissected out and trypsinized in 0.5 % trypsin and DNase at 37 °C for no more than 5 min. 0.2 mg/ml trypsin inhibitor was added to stop the trypsinization and tissues were shattered into single cells by pipetting. After removal of trypsin solution, cells were seeded in fibronectin coated plates with NPC medium (DMEM F-12/1 % Penn-Strep/1 % L-glutamine/1 % N-2 supplement/bFGF (10–20 ng/ml)). To induce NPC differentiation, FGF2 was removed from the culture medium and 1 μM retinoic acid/1 % FBS was added to the medium for 7 days.

### DNA construct, Transfection and Lentivirus production

Human RBM8a cDNA was PCR amplified and cloned into the lentiviral vector pLV-3FLAG3HA-T2A-GFP as previously described [53] and pTRIPZ-mCherry to generate the fusion gene, pTRIPZ-mCherry-RBM8a. The sequences for shRNAs targeting mouse RBM8a are as follows: control shRNA: 5′-CGGCTGAAACAAGAGTT GG-3′; shRNA-1: 5′- GCGGACCTTGTGT TTATATTT-3′; shRNA-2: 5′- CCATGACAAATTCG CTGAATA -3′; in lentiviral pLKO.1 vector (Sigma).

Transfection was performed according to a published protocol using polyethylenimine [84]. Mouse CAD cells were plated in a 24-well plate on coverslips. Control or RBM8a shRNAs (0.5 μg) were cotransfected with a GFP reporter (0.1 μg). To determine the knockdown effect of RBM8a, cells were fixed in 4 % paraformaldehyde 48 h posttransfection and stained with RBM8a antibody.

To generate stable SH-SY5Y cell line, pTRIPZ-mCherry or pTRIPZ-mCherry-RBM8a were cotransfected with psPAX2, pCMV-VSVG, into 293 T cells using PEI method. VSVG-pseudotyped lentiviruses were collected 48 after transfection and concentrated by ultracentrifugation at 36,000 rpm for 90 min [85]. The lentiviruses expressing pTRIPZ-mCherry or pTRIPZ-mCherry-RBM8a were co-infected with virus expressing rtTA in SH-SY5Y cells at multiplicity of infection (MOI) =20. Stable clones were selected by puromycin selection.

### Antibodies and immunoblot

The primary antibodies used in this study were the following: rabbit anti-HA antibody, mouse anti-F-actin antibody, mouse anti-RBM8a antibody, goat anti-Sox2 antibody, goat anti-doublecortin (DCX) antibody, rabbit anti-TLE4 (Santa Cruz); chick anti-GFP antibody (Aveslabs), mouse anti-βIII tubulin antibody (Tuj1, Covance), rabbit anti-Ki67 antibody (Lab Vision), mouse anti-BrdU antibody (DAKO), rabbit anti-phospho-Histone H3 ser10 antibody (Millipore), rabbit anti-mCherry RFP antibody (GenScript), mouse anti-NLGN1, mouse anti-ATXN1 (NeuromAb); mouse anti-KIF1A (BD Bioscience). The secondary antibodies used in this study were obtained from Invitrogen and Jackson ImmunoResearch.

Immunoblots were performed as described previously [85]. The total proteins were prepared from NPCs, N2a cells, embryonic cortex at different ages (E9-18) and adult brain (2 months) using 500 μl cell lysis buffer. Cell lysis buffer contains 20 mM Tris–HCl (pH 7.5), 150 mM NaCl, 1 mM Na_2_EDTA, 1 mM EGTA, 1 % Triton, 2.5 mM sodium pyrophosphate, 1 mM β-glycerophosphate, 1 mM Na_3_VO_4_, 1 μg/ml leupeptin, 2 μg/ml aprotinin, 1 mM PMSF. After homogenization on ice for 10 min, the supernatant of each sample was collected and stored in aliquots at −70 °C. For each sample, the protein concentration was determined by Bradford assay (Thermo Fisher Scientific Inc.). 20–50 μg of cell lysate were resolved by 12 % SDS-polyacrylamide gel electrophoresis and transferred into PVDF membranes. The protein blots were blocked with 5 % milk in TBS (10 mM Tris-HCl,pH 8.0, 150 mM NaCl) overnight at 4 °C and incubated with anti-RBM8a antibody, anti-RFP antibody, anti-Tuj1 antibody, anti-Sox2 antibody, or anti-Actin antibody at a dilution of 1 to 1000 to 2000 in 5 % milk prepared in TBS. The secondary antibody was anti-mouse IgG or anti-rabbit IgG (Santa Cruz). Immunoreactivity was detected with an enhanced chemiluminescence detection kit according to the company’s instructions (ECL, Millipore).

### *In utero* electroporation, BrdU labeling, Immunohistochemistry

The pregnant female mouse was anesthetized by intraperitoneal (i.p.) injection of avertin (800 ul/40 g) and Buprenorphine (0.05 mg/kg). The anesthetized female was put on the sterile warm pad at 37 °C and eye ointment was applied to prevent eyes from drying. Control or RBM8a shRNA constructs together with an enhanced GFP (EGFP)-expressing plasmid (final concentration, 2 μg/μl; pCAGIG-Venus) at a 2:1 ratio were injected into the lateral ventricle of the embryonic brains at E13. In the overexpression experiments, vector or human WT-RBM8a plasmid was electroporated into E14 embryonic brains. The electric pulses were delivered through the brain of the embryos (5 pulses of 30–45 V). After electroporation, embryos were put back to original position. The mother mice were sutured and returned to animal facility for recovery. BrdU (100 mg/kg) was i.p. injected into mice 2 h before sacrifice. Brains at E16 were removed and post-fixed in 4 % paraformaldehyde at 4 °C for overnight.

Brain sections (10-μm thick) were blocked with 5 % normal donkey serum in PBS with 0.3 % Triton X-100 for 60 min and were incubated with primary antibodies, anti-GFP (1:1000), anti-BrdU (1:500), anti-Ki67 (1:500), anti-pH3 (1:500), or anti-Tuj1 (1:500) antibodies overnight at room temperature. After the wash step with PBS, sections were incubated for 1 h at room temperature with Alexa488 and Alexa560 labeled secondary antibodies (1:400, Invitrogen). Sections were then stained with TO-PRO®-3 DNA dye (Invitrogen) and washed with PBS three times, and mounted with Anti-fade solution. The distribution of GFP positive cells was determined by dividing the number of GFP positive cells in each layer by the total number of GFP-positive cells in the entire section. In the cell cycle exit experiments, brains were harvested 24 h after BrdU injection. Brain sections were then co-stained with anti-GFP, anti-Ki67 and anti-BrdU antibodies. The cell cycle exit index was qualified by the ratio of GFP+/BrdU+/Ki67- to total GFP+/BrdU+ cells. Stereological quantification of stained cells within the neocortex was carried out as previously described [86].

### Image acquisition and statistical analysis

All images were acquired using a Zeiss LSM Pascal confocal microscope (Carl Zeiss, USA), and slides were analyzed by Zeiss LSM image browser software (Carl Zeiss, USA), Adobe Photoshop and ImageJ v1.37. Statistical analysis was performed using the student *t*-test. All bar graphs are plotted as mean ± SEM. In all analyses, *p* < 0.05 was considered statistically significant.

### RNA-seq analysis

SH-SY5Y cells stably expressing RBM8a or a control vector were created as described in the previous section, and the total RNA was purified from the cells using TRIzol reagent. To preserve the noncoding RNAs, we used RiboZero Kit (Invitrogen) to remove the ribosomal RNA and RNAs were used for construction of a sequencing library using the Illumina TruSeq Stranded total RNA Kit (NIH NHLBI Sequencing Core). One lane of 50 × 50 paired-end reads were generated by the Illumina HiSeq 2500 platform at the NHLBI DNA Sequencing and Genomics Core (DSGC) and mapped to the human genome (UCSC build hg19) using Tophat [87]. Gene type information for each gene was obtained from GRCh37.75 annotation [88].

Cuffdiff (http://cufflinks.cbcb.umd.edu/) was used with the reference genomes to identify the differentially expressed genes and isoforms [87]. The fold change for each gene was determined by dividing the FPKM for each gene in the overexpression sample, by the FPKM of the control sample. The cutoff of genes considered to be differentially expressed was a q-value of 0.05 or less, and either greater than a 1.5 fold upregulation or less than a 0.67 fold downregulation.

The list of differentially expressed genes was cross-referenced with databases consisting of genes known to be associated with various neurological disease. The number of genes present in both datasets was determined, and a hypergeometric analysis was used to identify the statistical probability that the genes in common were due to chance (data presented had a *p* < 0.05).

The list of common genes generated in the cross-reference with disease databases was then input in to DAVID gene functional classification tool (functional annotation cluster). DAVID identified what signaling pathways and what functional categories were significantly affected (Fisher exact test) based on the number of genes in the pathway located in the dataset.

For the alternative splicing analysis, all bam files created by TopHat (version 2.0.6) [89] were merged into a single files using samtools (version 1.1) [90]. The total number of reads that support the individual variants associated with each of the predicted functional alternative splicing events were determined using the MISO package (version 0.5.3) [91] using events annotated as of 26 June 2013.

Significant differentially spliced events were determined by requiring a Bayes’ factor >10 and Δψ >0.2 in a comparison of cherry and RBM8a. Each event was required to pass the default MISO minimum read coverage thresholds.

Transcripts targeted by NMD were determined by cross-referencing the list of differentially expressed RNAs with the hg19 annotation. Targets were identified as targeted by NMD if they had a 3′ UTR greater than or equal to 1250 bp, an intron in the 3′ UTR, or an exon junction greater than 50 nt downstream of a stop codon [74]. All RNAseq data has been submitted to Genbank (Bioproject PRJNA283786).

## Additional files

Additional file 1:
**Confirmation of RBM8a shRNAs** ***in vitro.*** **a.** Control or RBM8a shRNAs were transfected into CAD cells. 48 h posttransfection, protein lysates were probed with specific RBM8a mouse monoclonal antibody. **b.** Control or RBM8a shRNA2 was cotransfected with a GFP expressing construct in CAD cells. After 48 h, cells were stained with RBM8a antibody (red) and GFP (green) to show specific knockdown of endogenous RBM8a. White arrows indicated that RBM8a shRNA expressing cells express significantly low level of RBM8a compared to non-transfected cells (yellow arrowhead) in the same field. Additional file 2:
**RBM8a regulates cell proliferation** ***in vitro.*** **a.** Control or RBM8a shRNA2 was cotransfected with a GFP expressing construct in CAD cells. After 48 h, cells were pulse labeled with 10 μM BrdU for 30 min and stained with BrdU antibodies. Graphs show the percentage of GFP/BrdU double positive cells in each condition. ***, *p* < 0.001; *n* = 3. *t*-test. **b.** Control or RBM8a shRNA2 was cotransfected with a GFP expressing construct in CAD cells. After 48 h, cells were fixed with 4 % paraformaldehyde and stained with pH3 antibodies. Graphs show the percentage of GFP/ pH3 double positive cells in each condition. *, *p* < 0.05; *n* = 3. *t*-test. **c.** Vector or RBM8a overexpression plasmid was cotransfected with a GFP expressing construct in CAD cells. After 48 h, cells were pulse labeled with 10 μM BrdU for 30 min and stained with BrdU antibodies. Graphs show the percentage of GFP/BrdU double positive cells in each condition. *, *p* < 0.05; *n* = 3. *t*-test. **d.** Vector or RBM8a overexpression plasmid was cotransfected with a GFP expressing construct in CAD cells. After 48 h, cells were fixed with 4 % paraformaldehyde and stained with pH3 antibodies. Graphs show the percentage of GFP/ pH3 double positive cells in each condition. *, *p* < 0.05; *n* = 3. *t*-test. Additional file 3:
**RBM8a regulates cell migration** ***in vivo.*** **a.** Confocal images of E18 brain sections showing cell positioning after *in utero* electroporation of shRNA constructs at E15. The graph shows that knockdown of RBM8a significantly decreased the number of GFP-positive cells in the VZ/SVZ and increased the cell number in the CP. **b.** E16 brain sections staining with GFP and Tuj1 (red) show cell positioning after *in utero* electroporation of RBM8a shRNAs construct at E13. **C.** E16 brain sections staining with GFP and Tuj1 (red) show cell positioning after *in utero* electroporation of an overexpression construct at E14. Additional file 4:
**RNAseq quality of RBM8a stable RBM8a overexpression line.** **a.** Stable SH-SY5Y cells stably expressing mCherry or RBM8a-mCherry gene were confirmed by Western blotting by anti-mCherry antibody. **b.** Illumina Hi-Seq per base sequence quality for SH-SY5Y cells stably expressing mCherry. **c.** Illumina Hi-Seq per base sequence quality for SH-SY5Y cells stably expressing RBM8a-mCherry. Additional file 5: Table S1. Percentage of different types of RNAs. Additional file 6: Table S2. RBM8a regulates Genes that are involved in risks of diseases. Additional file 7: Table S3. Functional Clusters of RBM8a downstream genes. Additional file 8: Table S4. List of alternatively spliced protein coding RNAs. Additional file 9: Table S5. RBM8a mediated alternative splicing genes significantly overlap with ASD risk genes. Additional file 10: Table S6. Differentially expressed RNAs that are potential NMD targets. Additional file 11:
**Functional clusters of overlapping genes from potential RBM8a mediated NMD targets and ASD risks.** **a.** Number of genes that overlaps between potential NMD targets and ASD risk. **b.** Functional clusters of the overlapping genes. Additional file 12: Table S7. RBM8a mediated potential NMD targets significantly overlap with risk genes for neurological disease.

## Abbreviations

AD: Alzheimer’s disease
ASD: Autism spectrum disorder
CNV: Copy number variation
CP: Cortical plate
DCX: Doublecortin
EJC: Exon junction complex
ECM: Extracellular matrix
ID: Intellectual disability
IZ: Intermediate zone
NMD: Nonsense-mediated mRNA decay
NPC: Neural progenitor cell
pH3: Phospho-histone H3
PTC: Pre-termination stop codon
SCZ: Schizophrenia
TAR: Thrombocytopenia with absent radius
VZ/SVZ: Ventricular zone/subventricular zone
SE: Skipped exon
A5SS: Alternative 5′ splice site
A3SS: Alternative 3′ splice site
ALE: Alternative last exon
