# Supplementary figures and images for: A critical role of RBM8a in proliferation and differentiation of embryonic neural progenitors
Source: Neural Dev. 2015 Jun 21;10:18. doi: 10.1186/s13064-015-0045-7 (PMC4479087; doi:10.1186/s13064-015-0045-7)

## Additional File 1

A

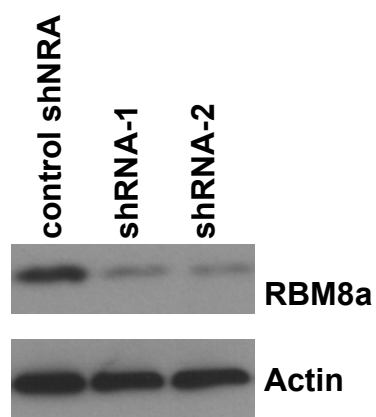

B

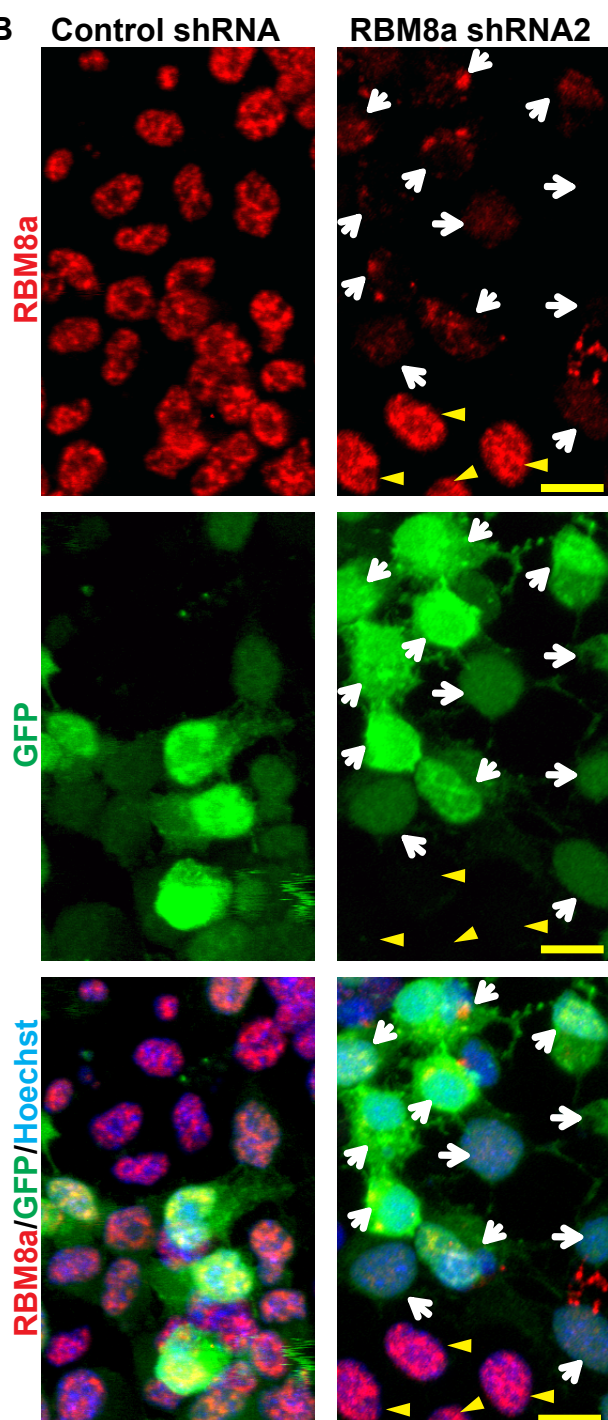

scale=10 um

Supplement: Additional file 1: — Confirmation of RBM8a shRNAs in vitro. a. Control or RBM8a shRNAs were transfected into CAD cells. 48 h posttransfection, protein lysates were probed with specific RBM8a mouse monoclonal antibody. b. Control or RBM8a shRNA2 was cotransfected with a GFP expressing construct in CAD cells. After 48 h, cells were stained with RBM8a antibody (red) and GFP (green) to show specific knockdown of endogenous RBM8a. White arrows indicated that RBM8a shRNA expressing cells express significantly low level of RBM8a compared to non-transfected cells (yellow arrowhead) in the same field. [file 13064_2015_45_MOESM1_ESM.pdf]

Additional File 2

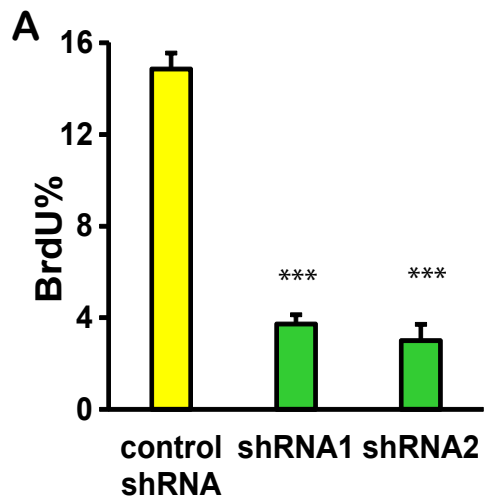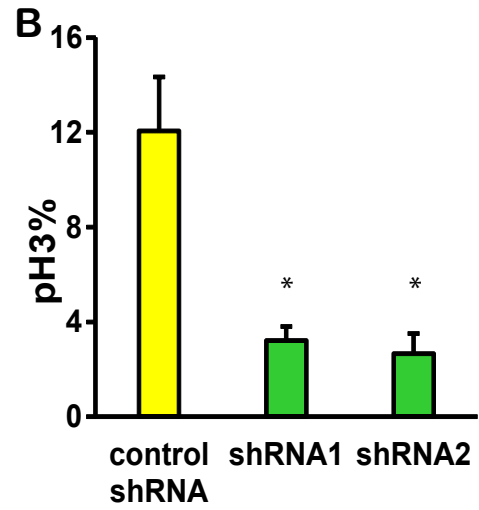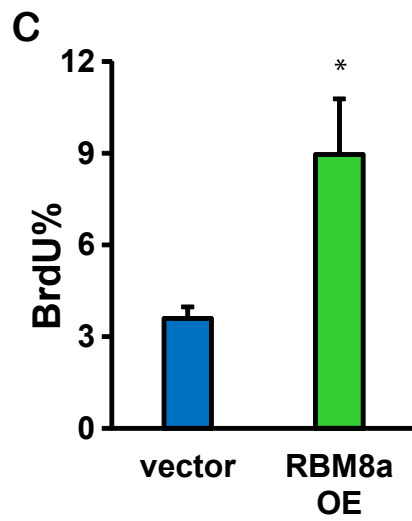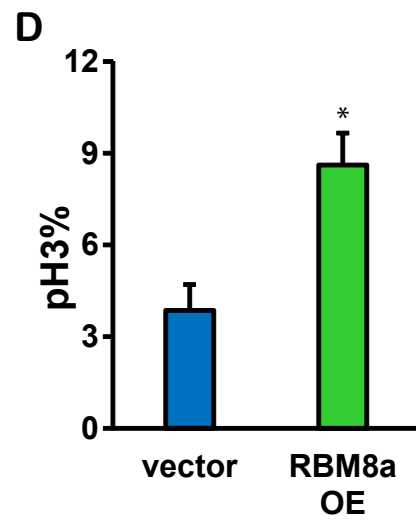

Supplement: Additional file 2: — RBM8a regulates cell proliferation in vitro. a. Control or RBM8a shRNA2 was cotransfected with a GFP expressing construct in CAD cells. After 48 h, cells were pulse labeled with 10 μM BrdU for 30 min and stained with BrdU antibodies. Graphs show the percentage of GFP/BrdU double positive cells in each condition. ***, p < 0.001; n = 3. t-test. b. Control or RBM8a shRNA2 was cotransfected with a GFP expressing construct in CAD cells. After 48 h, cells were fixed with 4 % paraformaldehyde and stained with pH3 antibodies. Graphs show the percentage of GFP/ pH3 double positive cells in each condition. *, p < 0.05; n = 3. t-test. c. Vector or RBM8a overexpression plasmid was cotransfected with a GFP expressing construct in CAD cells. After 48 h, cells were pulse labeled with 10 μM BrdU for 30 min and stained with BrdU antibodies. Graphs show the percentage of GFP/BrdU double positive cells in each condition. *, p < 0.05; n = 3. t-test. d. Vector or RBM8a overexpression plasmid was cotransfected with a GFP expressing construct in CAD cells. After 48 h, cells were fixed with 4 % paraformaldehyde and stained with pH3 antibodies. Graphs show the percentage of GFP/ pH3 double positive cells in each condition. *, p < 0.05; n = 3. t-test. [file 13064_2015_45_MOESM2_ESM.pdf]

# Additional File 3

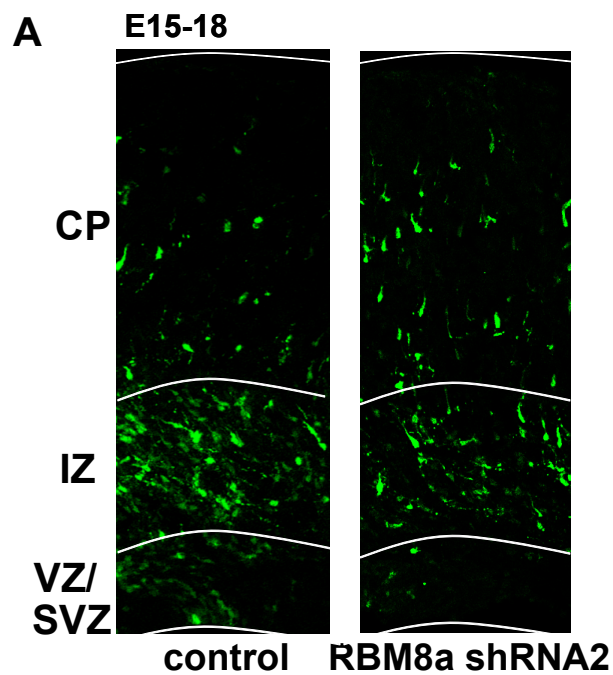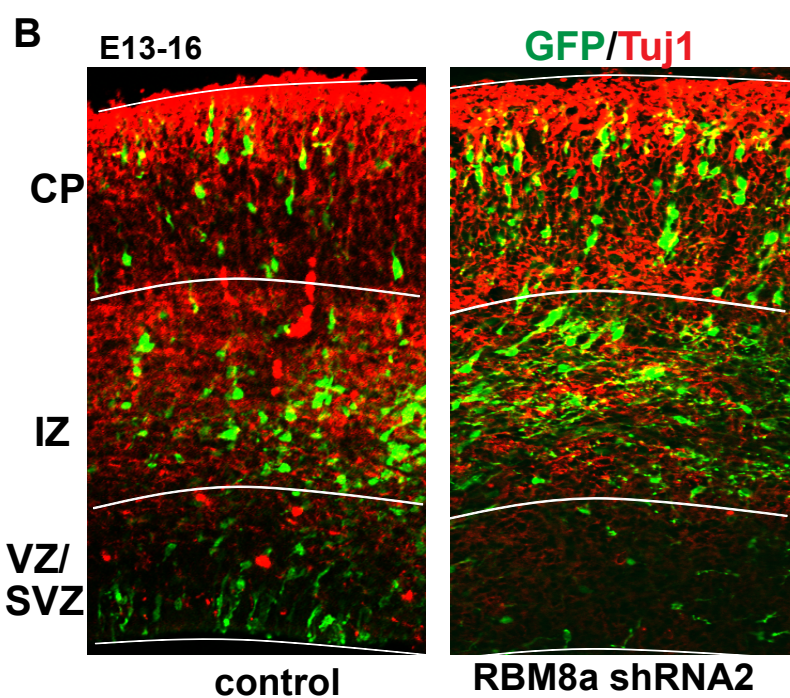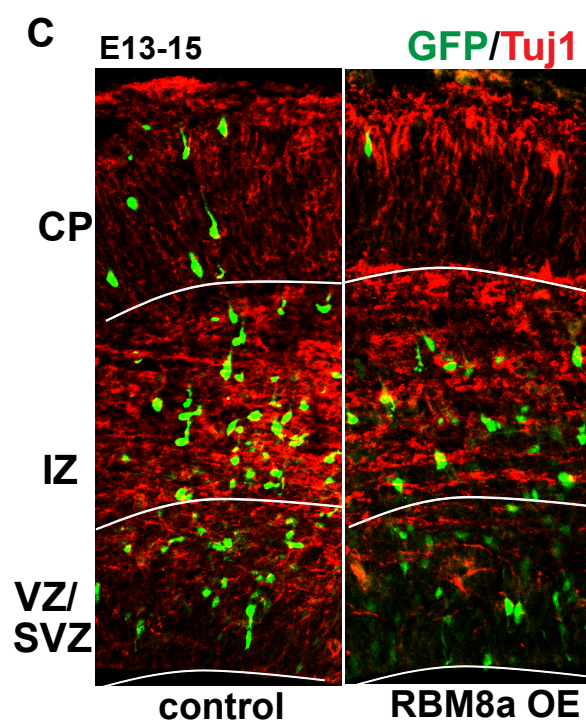

Supplement: Additional file 3: — RBM8a regulates cell migration in vivo. a. Confocal images of E18 brain sections showing cell positioning after in utero electroporation of shRNA constructs at E15. The graph shows that knockdown of RBM8a significantly decreased the number of GFP-positive cells in the VZ/SVZ and increased the cell number in the CP. b. E16 brain sections staining with GFP and Tuj1 (red) show cell positioning after in utero electroporation of RBM8a shRNAs construct at E13. C. E16 brain sections staining with GFP and Tuj1 (red) show cell positioning after in utero electroporation of an overexpression construct at E14. [file 13064_2015_45_MOESM3_ESM.pdf]

Additional File 4

A

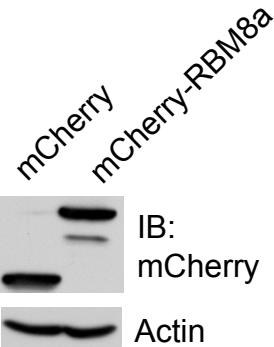

B

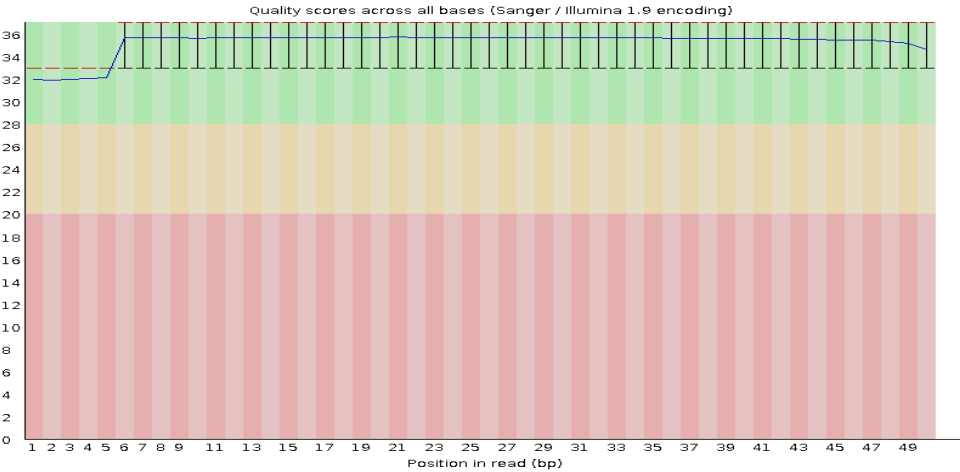

C

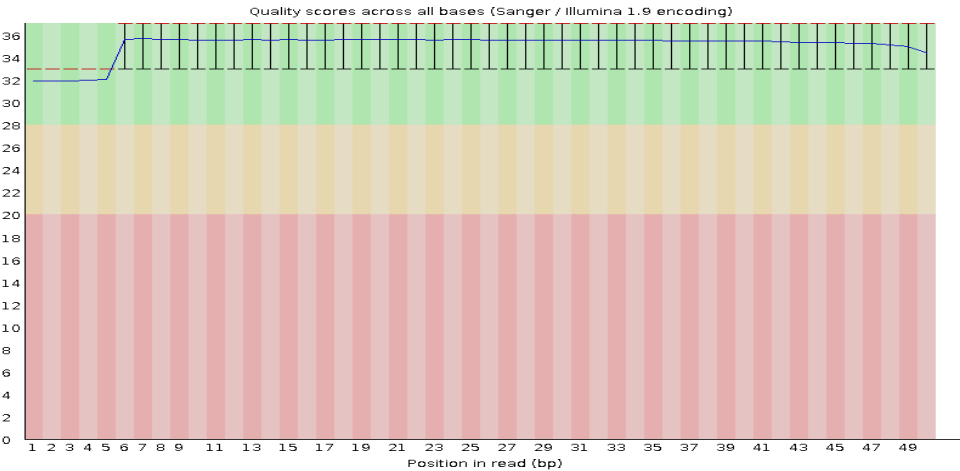

Supplement: Additional file 4: — RNAseq quality of RBM8a stable RBM8a overexpression line. a. Stable SH-SY5Y cells stably expressing mCherry or RBM8a-mCherry gene were confirmed by Western blotting by anti-mCherry antibody. b. Illumina Hi-Seq per base sequence quality for SH-SY5Y cells stably expressing mCherry. c. Illumina Hi-Seq per base sequence quality for SH-SY5Y cells stably expressing RBM8a-mCherry. [file 13064_2015_45_MOESM4_ESM.pdf]

**A**

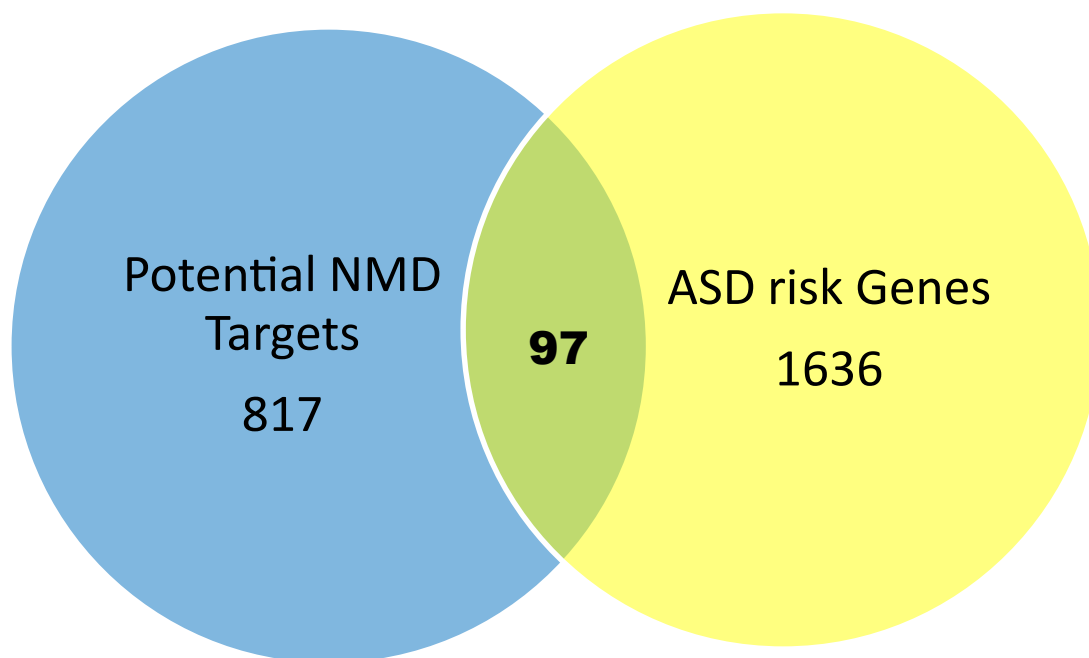

**B**

### Functions of potential NMD targets x ASD risk Genes

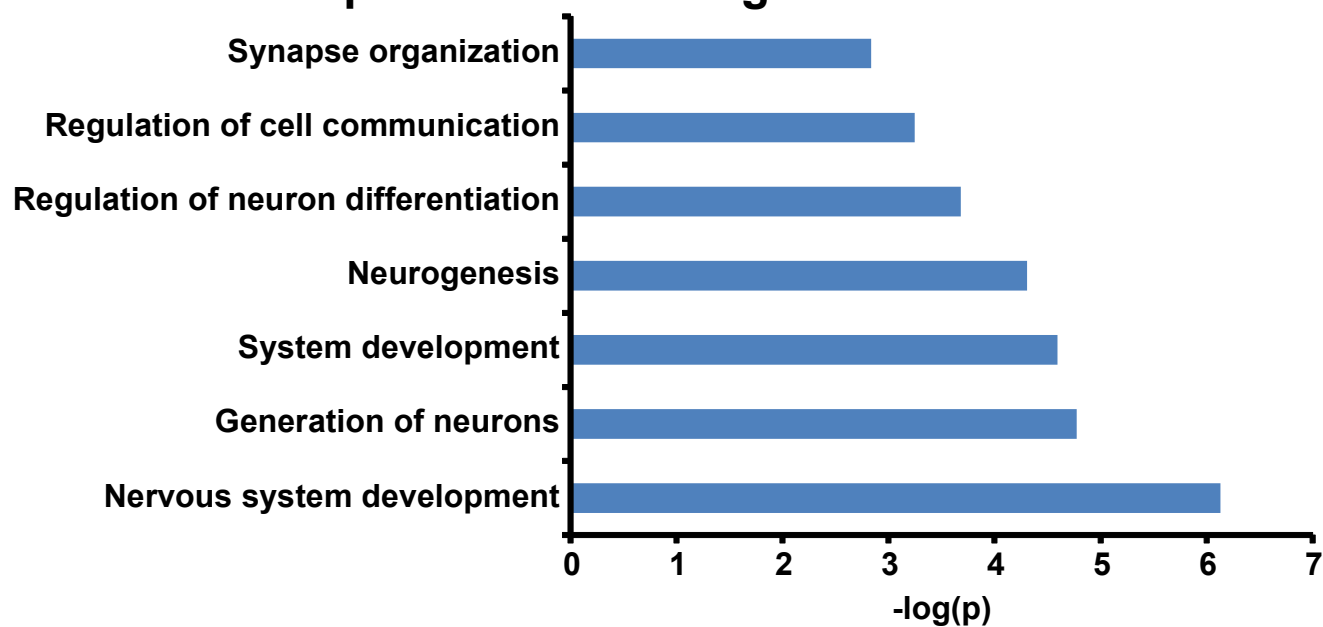

Supplement: Additional file 11: — Functional clusters of overlapping genes from potential RBM8a mediated NMD targets and ASD risks. a. Number of genes that overlaps between potential NMD targets and ASD risk. b. Functional clusters of the overlapping genes. [file 13064_2015_45_MOESM11_ESM.pdf]
