# Supplementary material for: A critical role of RBM8a in proliferation and differentiation of embryonic neural progenitors
Source: Neural Dev. 2015 Jun 21;10:18. doi: 10.1186/s13064-015-0045-7 (PMC4479087; doi:10.1186/s13064-015-0045-7)
Supplement: Additional file 5: Table S1. — Percentage of different types of RNAs. [file 13064_2015_45_MOESM5_ESM.pdf]

Additional File 5- Percentage of different types of RNAs

Type of RNA	Percentage
antisense	1.16618
lincRNA	1.57434
processed transcript	0.87464
protein coding	94.6939
sense intronic	0.05831
sense overlapping	0.05831
snoRNA	0.05831
psuedogene	1.45773
polymorphic pseudogene	0.05831
