# Supplementary material for: A critical role of RBM8a in proliferation and differentiation of embryonic neural progenitors
Source: Neural Dev. 2015 Jun 21;10:18. doi: 10.1186/s13064-015-0045-7 (PMC4479087; doi:10.1186/s13064-015-0045-7)
Supplement: Additional file 6: Table S2. — RBM8a regulates Genes that are involved in risks of diseases. [file 13064_2015_45_MOESM6_ESM.pdf]

Additional File 6-RBM8a regulates Genes that are involved in risks of diseases

ASD	AD	ID	Schizophrenia	Crohn
A2M, ABCA12, ABI3BP, ACTA2, ADAMTS1, ADAMTS10, ADAMTS3, AHNAK2, ALDH1L2, ALK, ANGPTL2, ANK1, AP3B2, ATP2B2, ATP2B4, ATP7B, BAIAP2, BCAR3, BCH4, BRSK2, C17orf97, CACNA2D2, CACNA2D3, CADPS, CD14, CD9, CDHR3, CERK, CGNL1, CHRND, CIT, CMPK2, CUX2, DBH, DCLK3, DDB2, DDR2, DEPDC4, DEPDC7, ECI2, EFCAB6, EPHB3, EPHX2, EPPK1, ERMP1, ERV3-1, ETHE1, EXD3, EYS, FAM65C, FBLN7, FN1, FRAS1, GABRB3, GBA, GLI2, GPC5, GPRASP1, GPX8, GRB14, GRM7, GXYLT2, HLTF, HMHA1, HSPA8, ICA1, IL20RB, IQGAP2, IQGAP3, IQSEC3, IQUB, ISLR2, JPH3, KALRN, KCNA3, KCN1, KCNS3, KIF14, KIF1A, KIRREL2, KRT80, L3MBTL1, LDLR, LGR4, LHFPL3, LINGO2, LRP4, LTBP1, MANSC1, MAP3K1, MASP2, MDM2, MGAT5B, MICALCL, MKI767, MOV10, MRC2, MSH5, MYADML2, MYH10, MYO1E, MYT1L, NFASC, NGEF, NLGN1, NOS2, NOTCH3, NR2F1, NRXN2, NSUN7, NUAK1, OXCT1, PAPSS2, PAQR8, PAX5, PCDHB16, PDGFD, PEX5L, PHACTR1, PITPNM3, PLCD1, PLEKHA6, PLXNB2, PLXND1, PPM1D, PRKCA, PTGR1, PTPRG, PTPRK, PTPRM, PTPRN2, RAD21L1, RASSF5, RELN, REST, RIMS1, RIMS2, RPRD1A, RRP12, SCRIB, SEMA6A, SERPINB8, SFPQ, SGIP1, SH3RF3, SHANK2, SHOX2, SLC25A12, SLC8A3, SCLCO1C1, SLITRK5, SNTA1, SORBS2, SPATA13, SREBF2, STXBP3, SULF2, SYNE2, TAS2R10, TBC1D4, TBX18, TBX4, TCF7L1, TCP11L2, TECTA, TNIK, TPR, TSNARE1, TTK, UACA, UBTF, UFL1, UNC13A, UNC80, UPF1, VAV3, VPS13A, VWCE, ZC3H12B, ZMYND12,	A2M, ABCA1, ABCA12, ACE, ACHE, ACTA2, ADAM9, ADRA2A, APOE, ATXN1, BAG3, BCHE, BDNF, CAST, CCL2, CD14, CDK5R1, CDKN2A, CHRNA3, CHRNB2, CSF1, CYP39A1, DBH, DRD4, EBF3, FAS, FBP1, FDPS, FGF1, FOS, GBA, GBP2, GRB14, GRIN3A, GSTO1, GSTT1, HMMR HSPG2, HTR6, IRF6, LIPA, MAPK8IP1, MAPT, MME, NEDD9, NGFR, NOS1, NRG1, NTRK2, PCK2, PLCE1, PPARGC1A, PTPLA RASSF4, RELN, RXRA, S100B, SEPT3, SLIT1, SNCA, SORL1, TAP1, TCF7L2, TCN2, VLDLR	ALK, ATP7B, CUX2, DBH, ERMP1, FRAS1, GPRASP1, GRB14, KCNA3, LHFPL3, MAP3K1, MGAT5B, MYO1E, MYT1L, NGEF, PHACTR1, SREBF2, UBTF, ZMYND12, ZNF540	ATP2B4, BAIAP2, CACNA1B, CACNA2D2, CACNB4, CAMK2B, CD14, DPYD, EPHX2, ESAM, LRRC48, MAN2A1, NCAN, NGEF, SLC8A1, TLE3, TSNARE1, ZNF804A	BSN, CCL2, IKZF1, REL, TNFSF4, ZFP36L1

ZNF117, ZNF404, ZNF540, ZNF804A				
