# Supplementary material for: A critical role of RBM8a in proliferation and differentiation of embryonic neural progenitors
Source: Neural Dev. 2015 Jun 21;10:18. doi: 10.1186/s13064-015-0045-7 (PMC4479087; doi:10.1186/s13064-015-0045-7)
Supplement: Additional file 7: Table S3. — Functional Clusters of RBM8a downstream genes. [file 13064_2015_45_MOESM7_ESM.pdf]

Additional File 7-Functional Clusters of RBM8a downstream genes

Function	Number of genes	*p* value	*q* value
Regulation of system processes	60	1.9×10^{-9}	6.9×10^{-6}
Neuron differentiation	75	4.9×10^{-9}	9.2×10^{-6}
Regulation of cell proliferation	114	9.4×10^{-9}	1.2×10^{-5}
Extracellular structure organization	38	2.0×10^{-8}	1.8×10^{-5}
Biological adhesion	101	9.8×10^{-8}	7.3×10^{-5}
Skeletal system development	56	2.4×10^{-7}	1.3×10^{-4}
Cell motion	74	3.3×10^{-7}	1.5×10^{-4}
Cell-cell signaling	88	3.4×10^{-7}	1.4×10^{-4}
Localization of the cell	53	9.0×10^{-7}	3.1×10^{-4}
Transmission of nerve impulse	57	2.2×10^{-6}	7.0×10^{-4}
Regulation of synaptic transmission	30	2.6×10^{-6}	7.0×10^{-4}
Regulation of cell development	39	3.0×10^{-6}	9.0×10^{-4}
Regulation of neurological system process	32	3.7×10^{-6}	9.1×10^{-4}
Neuron development	55	3.9×10^{-6}	9.0×10^{-4}
Regulation of neurogenesis	33	7.7×10^{-6}	1.7×10^{-3}
Synaptic transmission	49	9.8×10^{-6}	2.0×10^{-3}
Positive regulation of cell proliferation	62	1.2×10^{-5}	2.4×10^{-3}
Kidney development	23	1.2×10^{-5}	2.3×10^{-3}
ECM organization	24	1.5×10^{-5}	2.5×10^{-3}
Cell morphogenesis-differentiation	42	1.5×10^{-5}	2.4×10^{-3}
Response to inorganic substance	37	1.9×10^{-5}	2.8×10^{-3}
Response to corticosteroid stimulus	21	2.0×10^{-5}	2.9×10^{-3}
Enzyme linked receptor protein pathway	53	2.2×10^{-5}	3.1×10^{-3}
Neuron projected development	43	2.2×10^{-5}	3.0×10^{-3}
Forebrain development	30	2.5×10^{-5}	3.2×10^{-3}
Regulation of nervous system development	35	2.7×10^{-5}	3.3×10^{-3}
Urogenital system development	24	3.8×10^{-5}	4.6×10^{-3}
Vasculature development	41	6.7×10^{-5}	7.8×10^{-3}
Axongenesis	34	7.2×10^{-5}	7.9×10^{-3}
Blood vessel development	40	8.5×10^{-5}	9.0×10^{-3}
Neuron projection morphogenesis	36	1.0×10^{-4}	1.0×10^{-2}
Response to oxygen levels	27	1.2×10^{-4}	1.2×10^{-2}
Regulation of locomotion	33	1.5×10^{-4}	1.4 x 10-2
Diencephalon development	12	1.6×10^{-4}	1.5×10^{-2}
Sensory organ development	37	2.0×10^{-4}	1.8×10^{-2}
Response to glucocorticoids stimulus	18	2.2×10^{-4}	1.9×10^{-2}
Behavior	63	2.6×10^{-4}	2.1×10^{-2}
receptor tyrosine kinase pathway	36	2.8×10^{-4}	2.3×10^{-2}

Response to hypoxia	25	3.3×10^{-4}	2.6×10^{-2}
Response to steroid hormone stimulus	32	3.3×10^{-4}	2.6×10^{-2}
Morphogenesis of branching structure	17	3.7×10^{-4}	2.8×10^{-2}
Regulation of cell migration	29	4.2×10^{-4}	3.0×10^{-2}
Cell part morphogenesis	39	4.4×10^{-4}	3.1×10^{-2}
Regulation of cell proliferation	50	6.1×10^{-4}	4.2×10^{-2}
Platelet derived growth factor pathway	8	6.1×10^{-4}	4.2×10^{-2}
Adult behavior	18	7.5×10^{-4}	5.0×10^{-2}
Regulation of synaptic plasticity	15	7.5×10^{-4}	4.9×10^{-2}
