# Supplementary material for: A critical role of RBM8a in proliferation and differentiation of embryonic neural progenitors
Source: Neural Dev. 2015 Jun 21;10:18. doi: 10.1186/s13064-015-0045-7 (PMC4479087; doi:10.1186/s13064-015-0045-7)
Supplement: Additional file 8: Table S4. — List of alternatively spliced protein coding RNAs. [file 13064_2015_45_MOESM8_ESM.pdf]

Additional File 8-Alternative splicing protein coding RNAs list*

A3SS	A5SS	ALE	SE	AFE
ACBD3, BC038542, DDB2, GAK, GATAD2A, GLE1, MED1, MTHFD1L, NTSR1, PLK1S1, QRICH1, STK33, WNT2B	ARPC4, ARPC3-TTLL3, ATP13A1, BTAF1, C9orf24, CELSR3, FBXL20, LOC100130987, MAPK8IP3, MIR3134, NEO1, PHOSPHO2-KLHL23, PTBP3, RILPL1, SYS1, SYS1-DBNDD2, TRPC4AP		ACACA, AFF1, ARMC6, ATP13A1, CARD8, DBN1, DST, EML3, FHAD1, GAK, KCNN3, KCNQ5, KDM6B, MCOLN3, PTPRS, RAPGEF6, RECQL5, RPF2, SDK2, SLC35E2, SLC35E2B, SNAP91, ST3GAL2, TRIM16, USP36, VWA5A, WASF2, WHSCF1, ZFPM1, ZNF561	ABI1, ACOT7, ADAM2[illegible] ARFGEF1, ARHGAP10, [illegible] CC2D2A, CELSR3, CH[illegible] DNHD1, ELMO2, ERC1[illegible] GCNT2, GRAMD4, GS[illegible] IFFO1, IGF2BP2, IKZF2[illegible] LMO7, MDM2, MSH5-S[illegible] MVB12B, NDUFAF6, N[illegible] NRG1, NRXN1, NT5DC[illegible] RNF220, ROR2, SARDH[illegible] SMEK2, SPATA6L, ST3[illegible] STXBP1, TCF7L1, TME[illegible] VGLL4, ZNF280D, ZNF[illegible]

***This table notates only the protein coding RNAs that were alternatively spliced. Additional non-coding RNAs were found to be alternatively spliced, but are not reported in this table (such as ALE events).**
