# Supplementary material for: A critical role of RBM8a in proliferation and differentiation of embryonic neural progenitors
Source: Neural Dev. 2015 Jun 21;10:18. doi: 10.1186/s13064-015-0045-7 (PMC4479087; doi:10.1186/s13064-015-0045-7)
Supplement: Additional file 9: Table S5. — RBM8a mediated alternative splicing genes significantly overlap with ASD risk genes. [file 13064_2015_45_MOESM9_ESM.pdf]

Additional File 9- RBM8a mediated alternative splicing genes significantly overlap with ASD

Disease	*p*-value
ASD	7.57×10^{-7}
SCZ	0.326
AD	0.577
ID	0.174
Crohn's Disease	No common genes
