# Supplementary material for: A critical role of RBM8a in proliferation and differentiation of embryonic neural progenitors
Source: Neural Dev. 2015 Jun 21;10:18. doi: 10.1186/s13064-015-0045-7 (PMC4479087; doi:10.1186/s13064-015-0045-7)
Supplement: Additional file 10: Table S6. — Differentially expressed RNAs that are potential NMD targets. [file 13064_2015_45_MOESM10_ESM.pdf]

Additional File 10- Differentially expressed RNAs that are potential NMD targets

Long 3' UTR (> 1250 bp)	Intron in the 3' UTR	Exon junction > 50 nt downstream from a stop codon
ABAT, ABCA1, ABTB2, ACPP, ACSL6, ADAM9, ADAMTS1, ADAMTS3, ADAMTS9, ADRA2A, AEN, AFAP1, AHRR, AIF1L, AK4, AKAP12, ALDH1A3, ALDH1L2, ALDH4A1, ALPK1, ALPK3, AMOTL2, ANGPTL2, ANK1, ANKRD33B, ANO3, ANP32E, AP5Z1, APC2, APCDD1L, APOL4, APOLD1, ARC, ARHGAP24, ARHGAP29, ARHGAP42, ARHGEF6, ARID5B, ARRDC4, ARSJ, ASCC3, ASIC1, ASTN1, ATCAY, ATF3, ATP1B2, ATP2B2, ATP7B, ATP81A, ATXN1, B3GALT6, B3GALTL, B3GALTL, BAALC, BAHCC1, BAIAP2, BDNF, BEND6, BHLHE40, BLOC1S2, BNC2, BRSK2, BSN, BTG2, BTN3A2, C10orf32, C11orf70, C12orf5, C17orf51, C18orf54, C3orf14, C3orf52, C3orf62, C4orf25, C7, C8orf37, CABP7, CACNA1B, CACNA2D2, CALB1, CALCRL, CAMK2B, CAMK2N1, CAMKV, CAPN6, CAST, CBLN1, CCDC25, CCDC3, CCDC64, CCDC68, CCDC88C, CCND1, CCPG1, CCR6, CDC42EP4, CDHR1, CDHR3, CDK18, CDK5R1, CDKN1A, CECR6, CELF3, CELF4, CELF5, CELF6, CEP170B, CEP44, CERK, CERKL, CGNL1, CHCHD7, CHL1, CHN2, CHRNA3, CHRNB2, CHRND, CHST1, CHST15, CIT, CLIC2, CLMN, CLSTN2, CMPK2, CNR1, CNTN1, CNTN2, CNTNAP2, COBL, COBLL1, COL1A1, COL4A1, COL5A1, COLQ, CO17, CORIN, CPLX1, CPLX2, CPNE4, CSF1, CTBS, CUX2, CXorf38, CYB561, CYFIP2, CYS1, DAAM2, DAPK2, DCLK3, DCX, DDIT4L, DDN, DDX51, DDX60L, DGKI, DGKK, DIAPH2, DIEXF, DKK2, DLG3, DNAJC30, DNAJC5, DOCK3, DOCK5, DOK4, DOK6, DPP10, DRAM1, DSEL, DUSP4, DUSP8, EBF3, EDA2R, EDARADD, EDNRA, EDNRB, EFCAB1, EFNB1,	ADAM8, ADAMTS9, AKAP12, ALDH4A1, ANK1, ANP32E, ARC, ARBCF, ASB9, B2M, B3GAT1,BAIAP2, BCL2L12, BEND6, BRSK2, BSN, BST2, BTN3A2, C10orf32, C16orf95, C1orf54, C21orf58, C8orf31, CAMK2B, CAST, CDKN2A, CELF3, CELF4, CELF5, CELF6, CHCHD7, CLYBL, COX17, CSF1, CXorf38, DDC, DUSP28, EDA2R, EFCAB1, ENC1, FHIT, FLT3LG, FOXO1, FRMD4A, GCAT, GLB1L2, GLRX, GRIA4, GRM7, GRM8, HBEGF, HLA-E, HRAS, HRK, ID2, ID3, ID4, IGFBP3, INSIG1, JPH1, KCNC3, KRBOX4, LDB2, LHFPL5, LDB2, LHFPL5, LRRC23,	ADAM8, ADAMTS9, AKAP12, ALDH4A1, ANK1, ANP32E, ARC, ARBCF, ASB9, B2M, B3GAT1,BAIAP2, BCL2L12, BEND6, BRSK2, BSN, BST2, BTN3A2, C10orf32, C16orf95, C1orf54, C21orf58, C8orf31, CAMK2B, CAST, CDKN2A, CELF3, CELF4, CELF5, CELF6, CHCHD7, CLYBL, COX17, CSF1, CXorf38, DDC, DUSP28, EDA2R, EFCAB1, ENC1, FHIT, FLT3LG, FOXO1, FRMD4A, GCAT, GLB1L2, GLRX, GRIA4, GRM7, GRM8, HBEGF, HLA-E, HRAS, HRK, ID2, ID3, ID4, IGFBP3, INSIG1, JPH1, KCNC3, KRBOX4, LDB2, LHFPL5, LDB2, LHFPL5, LRRC23, MEIS1, MICAL2, MMP28, MND1, MORN5, NRG1, PBX1, PHLDA3, PLCE1, PLCH1, PLEKHA5, PLEKHA6, PZLC3, PROM1, PTPRO, PWWP2B, RAP1GAP, RBM3, RBPMS2, RGS7, SCN3B, SKAP2, SLC29A3, SPECC1, STMN4, SYP, TAC3, TCFL2, TMEM63A, TOM1L1, TPI1P2, ULBP1, UPF1, VMO1, YBX2,

EFNB2, ELAVL3, ELMOD1, ELOVL5, EMP2, ENC1, ENDOD1, ENPP6, EPB41L4B, EPHA10, EPHA8, EPN3, ERMP1, ERO1L, ESRRG, ETV1, EYA4, EYS, F2RL1, FAM114A1, FAM124A, FAM129A, FAM132B, FAM134B, FAM135A, FAM13B, FAM13C, FAM155B, FAM212B, FAM46A, FAM71F2, FAM83H, FAS, FAXC, FBXO32, FBXO39, FERMT1, FGF1, FGF14, FGFRL1, FKBP5, FLCN, FLJ45513, FMN1, FNDC5, FOXC1, FOXO1, FOXR2, FRAS1, FREM1, FREM2, FRMD3, FRMD4A, FRMD4B, FRZB, FSTL1, FSTL3, FSTL5, FUT10, FUT8, FZD4, FZD7, GABRB3, GABRP, GAREM, GATA2, GBP2, GDAP1L1, GINS4, GJA1, GLI2, GOLGA7B, GPC4, GPD1, GPM6A, GPR124, GPR155, GPR176, GPR56, GPRC5B, GPX8, GRAMD4, GRHL1, GRIA4, GRIN3A, GRIP1, GYG2, H1F0, HBEGF, HEG1, HES2, HIF3A, HLA-E, HLTF, HMGXB4, HMHA1, HMP19, HNF4G, HPCAL4, HR, HS6ST3, HSPB7, HTR6, HYOU1, ID4, IFIT1, IGDCC3, IGF2BP2, IGFBP3, IGFBP5, IGLON5, IKZF1, IMPACT, INA, INADL, INSC, INSIG1, INTS6, IPO5P1, IQSEC3, IRF6, IRGQ, IRS2, ISLR2, ITGAV, ITIH5, ITPKB, ITPR2, JAG1, JAG2, JAM2, JAZF1, JDP2, JPH1, JPH3, JRKL, KALRN, KAT2B, KCNC1, KCNG3, KCNJ2, KCNJ9, KCNK3, KCNMB2, KIAA0825, KIAA1024, KIAA1217, KIAA1377, KIAA1549L, KIAA1958, KIF14, KIF1A, KIF21B, KIRREL, KLF10, KLF11, KLF9, KLHL14, KRBOX4, KRT80, L3MBTL3, LAMC1, LANCL2, LBH, LDB2, LDB3, LDLR, LDLRAD4, LEPR, LGR4, LHFPL3, LHFPL4, LIF, LIMCH1, LIPA, LMO3, LM04, LOC100130705, LOC653602, LRP1B, LRP4, LSAMP, LURAP1L, LYNX1, LZTS1, MAK, MAN2A1, MANEAL, MAP2, MAP3K1, MAP3K14, MAP6, MAP7, MAPT, MB21D2, MBOAT1, MDM2, MEIS1, MEX3B, MFSD4, MFSD8, MGLL, MICAL2, MITF, MKI67, MLF1, MLLT3, MLLT4, MMAA, MME, MMP24, MPPED2, MPV17L,	MEIS1, MICAL2, MMP28, MND1, MORN5, NRG1, PBX1, PHLDA3, PLCE1, PLCH1, PLEKHA5, PLEKHA6, PZLC3, PROM1, PTPRO, PWWP2B, RAP1GAP, RBM3, RBPMS2, RGS7, SCN3B, SKAP2, SLC29A3, SPECC1, STMN4, SYP, TAC3, TCFL2, TMEM63A, TOM1L1, TPI1P2, ULBP1, UPF1, VMO1, YBX2, ZDHHC11, ZDHHC15, ZFP36L1, ZNF217, ZNF418	ZDHHC11, ZDHHC15, ZFP36L1, ZNF217, ZNF418

MRAP2, MST4, MTL5, MTUS1, MVB12B, MYCL, MYH10, MYLIP, MYO10, MYO1E, MYT1, MYT1L, NANOS1, NAT8L, NAV1, NCAN, NCAPG, NCOA5, NDST3, NEDD4, NEDD9, NEGR1, NEURL1, NFASC, NFATC4, NGFR, NHS, NKD1, NLGN1, NMNAT2, NOS1, NOTCH2, NPTXR, NPY2R, NRBP2, NRCAM, NRG1, NSG1, NT5E, NTRK2, NUAK1, NUDT10, NYAP2, OLFM3, OPN3, OXCT1, PAM, PAPSS2, PAQR5, PAQR8, PARP11, PATZ1, PAX5, PBX1, PCDH18, PCDHB16, PCTP, PDCD4, PDE11A, PDE8B, PDGFC, PDGFD, PDGFRA, PDGFRB, PDK1, PDK4, PEAR1, PER2, PER3, PEX5L, PFKFB2, PGBD5, PHEX, PHF21B, PHKA1, PHKB, PHYHIPL, PI4K2B, PID1, PIFO, PIGN, PITPNC1, PITPNM3, PKP2, PLCH1, PLD1, PLD6, PLEKHA2, PLEKHA6, PLEKHA7, PLEKHG4B, PLEKHH2, PLS3, PLXNA2, PLXNA2, PLXNA4, PMEPA1, PNMA3, PNMAL2, POC1B, POLD3, POU3F2, POU4F1, PPAP2B, PPARGC1A, PPFIA4, PPFIBP1, PPM1D, PP1R16B, PPP2R2C, PRDM11, PRICKLE1, PRKCA, PROX1, PRPS2, PRR11, PRR18, PRRT2, PRSS23, PRTFDC1, PTCHD4, PTER, PTGFR, PTGFRN, PTGR2, PTP4A3, PTPN14, PTPRB, PTPRE, PTPRG, PTPRK, PTPRN2, PTPRO, PVRL4, QKI, RAB11FIP4, RAB27A, RAB31, RABGAP1L, RALGPS2, RASSF4, RASSF5, RASSF8, RAX, RBM24, RBM3, RCN1, RD3, REL, RERG, REST, RET, RGL1, RGMB, RHOB, RIMKLA, RIMS1, RIMS2, RIMS4, RIN2, RNASEH2C, RPRD1A, RPS6KL1, RREB1, RUNX1T1, RXRA, SARM1, SCAMP5, SCG3, SCML1, SCN3A, SCN3B, SCN4B, SCN9A, SCRT1, SDC2, SDC3, SEMA3A, SEMA5A, SEMA6A, SERPINB8, SERPINB9, SERTAD2, SEZ6L, SFRP1, SGIP1, SH3BP5, SH3RF3, SHANK2, SHOX2, SIX1, SIX3, SIX4, SKAP2, SLC12A7, SLC16A12, SLC1A2, SLC25A12, SCL25A34, SLC29A3, SLCA12, SLCA4, SLC35F3, SLC43A2, SLC47A1, SLC6A2, SLC7A11, SLC7A14, SLCA5P1,		

SLC8A1, SCL8A2, SLC8A3, SLCO4C1, SLIT1, SLITRK5, SMG7, SMIM17, SMOC1, SMPD3, SNCA, SORBS2, SORL1, SOX4, SOX7, SOX9, SPARC, SPATA13, SPATA18, SPATAS2L, SPECC1, SPOCK1, SPOCK3, SPON1, SPRYD4, SPSB1, SRC, SREBF2, SRGAP2, SRRM3, SRRM4, ST8SIA3, ST8SIA3, ST8SIA4, STC2, STMN2, STMN3, STMN4, STON1, STS, STX1A, SULT4A1, SUSD5, SVOP, SYP, SYT13, SYT14, SYT2, SYT4, SYT7, SYTL5, TBC1D30, TBC1D4, TBX18, TCF7L2, TCTA, TEC, TFAP2B, TFPI, THRB, THSD4, TIMP3, TIPARP, TLE3, TLE4, TMCC3, TMEM104, TMEM105, TMEM108, TMEM123, TMEM151B, TMEM179, TMEM2, TMEME255A, TMEM35, TMEM56, TMEM63A, TMTC1, TNFRSF10B, TNFRSF19, TNFSF3, TNIK, TOR4A, TP53INP1, TPI1P2, TPPP, TPR, TRAF5, TRHDE, TRIM59, TRIM67, TRMT61A, TRPS1, TRPV1, TSHR, TSLP, TSNARE1, TSPAN18, TSPEAR, TTBK1, UACA, UAP1L1, UBASH3B, UBTF, UFL1, ULBP1, UNC13A, UNC5CL, UNC80, UPF1, USP25, VAV2, VAV3, VEGFA, VIPR2, VPS13A, VSIG10, WDFY2, WDR52, WDR77, WIPF1, WSB1, WWC2, WWTR1, XKR5, XYLB, YAP1, ZC3H12B, ZC3H14, ZDHHC15, ZDHHC22, ZFP3, ZFP35L1, ZFP3, ZFP36L1, ZFR2, ZMAT3, ZMAT4, ZNF117, ZNF124, ZNF135, ZNF208, ZNF217, ZNF319, ZNF334, ZNF418, ZNF488, ZNF528, ZNF578, ZNF662, ZNF667, ZNF677, ZNF764, ZSCAN31		
