# Supplementary material for: A critical role of RBM8a in proliferation and differentiation of embryonic neural progenitors
Source: Neural Dev. 2015 Jun 21;10:18. doi: 10.1186/s13064-015-0045-7 (PMC4479087; doi:10.1186/s13064-015-0045-7)
Supplement: Additional file 12: Table S7. — RBM8a mediated potential NMD targets significantly overlap with risk genes for neurological disease. [file 13064_2015_45_MOESM12_ESM.pdf]

Additional File 12- RBM8a mediated potential NMD targets are significantly involved in risks of diseases

**Disease**	***p*-value**
**ASD**	**4.12×10^{-9}**
**SCZ**	**6.32×10^{-5}**
**AD**	**1.16×10^{-3}**
**ID**	**1.30×10^{-2}**
**Crohn's Disease**	**0.232**
